# The *FAM3C* locus that encodes interleukin-like EMT inducer (ILEI) is frequently co-amplified in *MET*-amplified cancers and contributes to invasiveness

**DOI:** 10.1186/s13046-021-01862-5

**Published:** 2021-02-17

**Authors:** Ulrike Schmidt, Gerwin Heller, Gerald Timelthaler, Petra Heffeter, Zsolt Somodi, Norbert Schweifer, Maria Sibilia, Walter Berger, Agnes Csiszar

**Affiliations:** 1 Research Institute of Molecular Pathology, Dr. Bohr-Gasse 3, A-1030 Vienna, Austria; 2 Department of Medicine I, Division of Oncology, Medical University of Vienna, Währinger Gürtel 18-20, A-1090 Vienna, Austria; 3 Department of Medicine I, Institute of Cancer Research, Medical University of Vienna, Borschkegasse 8a, A-1090 Vienna, Austria; 4 Department of Oncology, Bacs-Kiskun County Teaching Hospital, Kecskemet, Hungary; 5 Present Address: Parexel International, 2 Federal St, Billerica, MA USA; 6 Boehringer-Ingelheim RCV GmbH & Co KG, Vienna, Austria

**Keywords:** Interleukin-like EMT inducer (ILEI), FAM3C, C-MET, Gene amplification, Invasion, Matrix metalloproteinase (MMP), Cancer

## Abstract

**Background:**

Gene amplification of *MET*, which encodes for the receptor tyrosine kinase c-MET, occurs in a variety of human cancers. High c-MET levels often correlate with poor cancer prognosis. Interleukin-like EMT inducer (ILEI) is also overexpressed in many cancers and is associated with metastasis and poor survival. The gene for ILEI, *FAM3C*, is located close to *MET* on chromosome 7q31 in an amplification “hotspot”, but it is unclear whether *FAMC3* amplification contributes to elevated ILEI expression in cancer. In this study we have investigated *FAMC3* copy number gain in different cancers and its potential connection to *MET* amplifications.

**Methods:**

*FAMC3* and *MET* copy numbers were investigated in various cancer samples and 200 cancer cell lines. Copy numbers of the two genes were correlated with mRNA levels, with relapse-free survival in lung cancer patient samples as well as with clinicopathological parameters in primary samples from 49 advanced stage colorectal cancer patients. ILEI knock-down and c-MET inhibition effects on proliferation and invasiveness of five cancer cell lines and growth of xenograft tumors in mice were then investigated.

**Results:**

*FAMC3* was amplified in strict association with *MET* amplification in several human cancers and cancer cell lines. Increased *FAM3C* and *MET* copy numbers were tightly linked and correlated with increased gene expression and poor survival in human lung cancer and with extramural invasion in colorectal carcinoma. Stable ILEI shRNA knock-down did not influence proliferation or sensitivity towards c-MET-inhibitor induced proliferation arrest in cancer cells, but impaired both c-MET-independent and -dependent cancer cell invasion. c-MET inhibition reduced ILEI secretion, and shRNA mediated ILEI knock-down prevented c-MET-signaling induced elevated expression and secretion of matrix metalloproteinase (MMP)-2 and MMP-9. Combination of ILEI knock-down and c-MET-inhibition significantly reduced the invasive outgrowth of NCI-H441 and NCI-H1993 lung tumor xenografts by inhibiting proliferation, MMP expression and E-cadherin membrane localization.

**Conclusions:**

These novel findings suggest *MET* amplifications are often in reality *MET-FAM3C* co-amplifications with tight functional cooperation. Therefore, the clinical relevance of this frequent cancer amplification hotspot, so far dedicated purely to c-MET function, should be re-evaluated to include ILEI as a target in the therapy of c-MET-amplified human carcinomas.

**Supplementary Information:**

The online version contains supplementary material available at 10.1186/s13046-021-01862-5.

## Background

Cancer develops via a complex multistep process [1]. Central to this are multiple genetic abnormalities that lead to disruption of the normal cell signaling networks. An example is when proto-oncogenes with physiological functions within healthy cells become hyperactive or dysregulated leading to uncontrolled cell growth. This can be the result of a mutation that switches an oncogene on permanently or from overexpression of the protein [2].

*MET* is a proto-oncogene that encodes a receptor tyrosine kinase (RTK), c-MET, a receptor for hepatocyte growth factor (HGF). c-MET is expressed in many epithelial cells, while HGF is secreted from mesenchymal cells. Their interaction stimulates various signaling pathways and has a vital role in breaking cell adhesions to promote motility of epithelial cells during embryogenesis and wound healing [3]. However, hyperactivity of c-MET is evident in a wide range of cancers where it can drive proliferation, survival, motility, and invasion [4]. In particular, high levels of c-MET often correlate with poor prognosis in cancer patients [4]. In some cancers, such as gastric and non-small cell lung carcinomas (NSCLCs), hyperactivity of c-MET is the result of multiple copies of the *MET* gene and these cells seem to be largely dependent on sustained c-MET activity for their growth and survival [3].

An increased gene copy number (CN) can result from gene amplification or aneuploidy. Gene amplification “hotspots” are mapped throughout the human genome for many cancers, several of them are also functionally linked to known oncogenes [5]. The *MET* gene is located on chromosome 7q31 in close proximity of another gene that has been implicated in cancer; the *FAM3C* locus is within 4.6 MB of the *MET* gene on chromosome 7q31. *FAM3C* encodes for the interleukin-like EMT inducer (ILEI) protein, a secreted factor that can regulate tumor progression [6]. *FAM3C* was identified as an epithelial-to-mesenchymal transition (EMT)-specific gene [7]. EMT occurs when epithelial cells lose their apical-basal polarity and cell-cell adhesion and switch to a migratory and invasive mesenchymal phenotype [8]. This process is vital during development and can be reactivated when required, such as during wound healing [9]. In cancer, EMT activities might be switched on transiently and reversibly to convert adherent epithelial tumor cells into motile and invasive mesenchymal cells [10]. In murine and human cellular models of breast, hepatocellular carcinoma, and lung cancer ILEI is required and sufficient to induce EMT and invasion in vitro and metastasis in vivo [6, 11, 12]. ILEI is overexpressed in several human tumors and shows altered subcellular localization, which is related to changes in the secretion levels of the protein [13]. ILEI localization strongly correlates with metastasis formation and survival in human breast and hepatocellular carcinomas [6, 11, 12]. In colorectal cancer, upregulation of ILEI protein expression correlates with EMT and poor prognosis [14].

However, whether increased expression of ILEI in some cancers is associated with increased CN of the *FAM3C* locus is unclear. The proximity of *FAM3C* to the *MET* hotspot suggests that the two genes may be often co-amplified. The aim of this study was to investigate whether increased *FAM3C* CN was evident in various cancers and reveal whether there was a relationship with *MET* amplification. Our results suggested a strong correlation between the copy number of the two genes. Further in vitro mechanistic investigations and xenograft experiments with combined counteraction of ILEI and c-MET activities suggest that c-MET and ILEI cooperate to increase the invasiveness of cancer cells.

## Methods

### Materials and methods

#### Cell lines

NCI-H1993 (CRL-5909), NCI-H441 (HTB-174), SKBR3 (HTB-30), MKN-45 (ACC409), OE33 (ACC706), MCF7 (HTB-22), MDA-MB-231 (HTB-26) and NIH3T3 (CRL-1658) cell lines were obtained from ATCC (http://www.lgcstandards-atcc.org/en.aspx) or DSMZ (http://www.dsmz.de/), tested for mycoplasma infection on a regular basis using a commercial biochemical test (Lonza) and authenticated using STR profiling. All cells were cultured in Dulbecco’s Modified Eagle’s Medium/Nutrient Mixture F-12 Ham, 1:1 mixture supplemented with 10% fetal calf serum (FCS), or in case of MKN45 with 20% FCS. Upon serum withdrawal, FCS was replaced by 0.1% bovine serum albumin.

#### Animals

Eight-twelve week-old female severe combined immunodeficiency disease (SCID) mice (Envigo, Italy) were used for xenograft experiments in this study. All animal work was done by following earlier protocols ethically approved by the Institutional Animal Care and Use Committee of the Medical University of Vienna and by the Austrian Bundesministerium für Bildung, Wissenschaft und Forschung (BMWFW-66.009/0081-WF/V/3b/2015).

#### Human tumor samples

Formalin fixed paraffin embedded primary tumor samples of advanced stage colorectal carcinoma were obtained from stored samples from de-identified patients treated in Kecskemet General Hospital, Hungary, who had previously provided informed consent for their use in clinical research.

#### Genomic DNA isolation and qPCR-based determination of gene CNs from formalin fixed paraffin embedded human tumor samples

Genomic DNA was isolated from non-stromal regions of 3–4 10 μm thick sections of formalin fixed paraffin-embedded tumors of 49 advanced-stage colorectal carcinoma patients using the Gentra Puregene Tissue Kit (Qiagen) according the manufacturer’s instructions. 60 ng of isolated genomic DNA was used as template in the quantitative real-time PCR reaction. All samples were done in triplicates and the *MET* and *FAM3C* copy numbers were derived by standardizing the input DNA to the control signal (*TOP3A*, chromosome 17p11) as described earlier [15]. The sequences of the primer pairs and probes for *TOP3A* and *MET* were as described in [16] using FAM as flourogenic label. For *FAM3C* the primers Hsp.FAM3C_F 5′-GTCACACTCTTGTGCCAGTCT-3′ and Hsp.FAM3C_R 5′-GAGCAAAGGTCAGGGTTGAAAG-3′ were used with the HEX-labeled probe Hsp.FAM3C_probe 5′-TCTGCAGCTTCAAATCCCCTCCTG-3′ allowing duplex PCR with the *TOP3A* control gene.

#### CN analysis of data in the TCGA database and human cancer cell lines

Gene CNs of *FAM3C*, *MET*, *EGFR*, and *FGFR1* were calculated using the genome sequencing datasets of several cancer entities in the TCGA database (https://www.cancer.gov/about-nci/organization/ccg/research/structural-genomics/tcga). GISTIC 2.0 values were obtained from the Firehose system using Caleydo v3 software (caleydo.org): − 2, deep deletion; − 1, shallow deletion; 0, diploid; 1, gain; 2, amplification. RNA-seq data were obtained from Cancer Browser database (https://genome-cancer.ucsc.edu/).

Copy number (CN) data of 200 tumor cell lines were generated using the GeneChip Human Mapping 250 K Nsp Arrays (Affymetrix) and subsequently analyzed on the Affymetrix® Genotyping Console™ software (GTC) using the unpaired CNAT 4.0 analysis algotrythms. Gene copy number ± 3 was considered as amplification, < 3 as non-amplified.

#### Generation of stable cell lines expressing ILEI shRNA vectors

For ILEI and mammalian non-targeting control shRNA knock-down in NCI-H1993, NCI-H441, MKN45, OE33 and SKBR3 cells, MISSION shRNA lentiviral transduction particles (Sigma, St Louis, MS, USA) were used according to the manufacturer’s instructions. Five shRNA sequences were pretested for ILEI knock-down (sh261 CCGGGATGCAAGTTTAGGAAATCTACTCGAGTAGATTTCCTAAACTTGCATCTTTTTG, sh328 CCGGCCAGATATAAGTGTGGGATCTCTCGAGAGATCCCACACTTATATCTGGTTTTTG, sh506 CCGGAGGAGAAGTATTAGACACTAACTCGAGTTAGTGTCTAATACTTCTCCTTTTTTG, sh579 CCGGGCCATACAAGATGGAACAATACTCGAGTATTGTTCCATCTTGTATGGCTTTTTG and sh1767 CCGGCCTGTGTTTATCTAACTTCATCTCGAGATGAAGTTAGATAAACACAGGTTTTTG) and two were selected (sh261 and sh506) as the most efficient for later studies. In studies with only one shILEI cell line, “shILEI” indicates sh506. Stable cell lines were established using selection for puromycin resistance of transduced cells. ILEI expression was validated in whole cell lysates and conditioned medium (CM) by Western blotting.

#### Western blot analysis

Western blot analysis was performed as previously described [13]. Cells were treated with crizotinib (500 nM), savolitinib (1 μM, both dissolved in DMSO) or DMSO for 24 h, conditioned media (CM) were collected after 24 h. Anti-ILEI [6], anti-phospho cMET (#3077), anti-cMET (#3127), anti-phosphoErk1/2, anti-Erk1/2, anti-E-cadherin, anti-vinculin (Cell Signaling Technologies), anti-α tubulin, and anti-β actin (Sigma) primary antibodies were used followed by enhanced chemiluminescent (ECL) detection using Chemidoc Touch (Bio-Rad) for digital capturing and ImageLab software (Bio-Rad) for visualization and quantification.

#### ^3^H-thymidine incorporation assay

Cells were pretreated with different concentrations of crizotinib for 24 h and seeded in triplicates in 96 well plates in the presence of the same inhibitor concentrations. After 24 h of incubation, cells were labeled with 30 μCi/ml methyl ^3^H-thymidine for 2 h. Radioactive media was removed, cells were washed in phosphate-buffered saline (PBS) and trypsinized. Cells were fixed by Tomtec cell harvester (Tomtec Inc., USA) onto a wax-embedded filtermat, and radioactive intensity was determined by a Wallac 1450 MicroBeta liquid scintillator (PerkinElmer Inc., USA). Results were normalized according to cell number.

#### MTT assay

Cells were plated in 100 μl complete medium in 96-well flat bottom plates in the presence or absence of crizotinib (500 nM) or vehicle (DMSO) or increasing concentrations of PHA665752 (Sigma) and Savolitinib (Selleckchem). After 24 h (crizotinib) or 72 h (PHA665752, savolitinib) of incubation 10 μl of 5 mg/ml 3-(4,5-dimethylthiazol-2-yl)-2,5-diphenyltetrazolium bromide (MTT) solution dissolved in PBS was added for 2 h followed by the addition of 100 μl of solubilization solution (40% dimethylformamide, 2% acetic acid, 16% sodium dodecyl sulfate, pH 4.7) and rigorous shaking to dissolve the formazan crystals. The absorbance at 570 nm was determined using a Tecan plate reader (Tecan, Austria). Triplicate wells were assayed for each condition.

#### Trans-well invasion assay

Cells were pre-starved overnight in starvation or low (1%) FCS medium and seeded in the same medium into trans-well invasion chambers with 8 μm pore size, coated with Matrigel (Corning Inc., USA) and pre-equilibrated with medium (50.000 cells/inlet, each condition in triplicates). The lower chamber of the trans-well unit contained conditioned medium of NIH3T3 cells or human HGF (40 ng/ml) as attractant. For c-MET inhibition, medium was supplemented with 500 nM crizotinib both in the upper and lower chambers. Cells were allowed to invade for 24 h, non-invaded cells were removed from the upper side of the inlets, and cells were fixed and stained with 4′,6-diamidino-2-phenylindole (DAPI). Total numbers of invasive cells were counted using fluorescent microscopy imaging followed by ImageJ analysis.

#### RNA isolation and real-time quantitative PCR analysis

Total RNA was extracted, reverse transcribed and cDNA was amplified with primers for the genes *MMP9*, *MMP2* and *CDH1* as described earlier [17].

#### Gelatin zymography

For MMP-9 and MMP-2 detection in the CM of NCI-H441 and NCI-H1993 shCont and shILEI cells upon HGF stimulus with or without crizotinib treatment cells were plated in 6-well plates. At 70–80% confluency, cells were crizotinib- (500 nM) or DMSO-treated for 30 min followed by medium change to FBS-free media with continued crizotinib supply and the addition of human HGF (40 ng/ml). 24-h CM were collected and concentrated as described for Western blot analysis and all samples were adjusted to the same protein concentration followed by equal volume gel loading.

For MMP-9 and MMP-2 detection in protein extracts, NCI-H441 and NCI-H1993 shCont and shILEI snap-frozen tumor pieces with or without crizotinib treatment were homogenized in lysis buffer (see Western blot analysis for composition), total protein concentration was determined and equal protein amounts were loaded.

A 7.5% acrylamide gel was prepared with 2 mg/ml final concentration of gelatin; samples were loaded in non-reducing sample buffer without boiling. After running, the gel was washed twice for 30 min each in washing buffer (2.5% Triton X-100, 50 mM TRIS-HCl, pH 7.5, 5 mM CaCl_2_, 1 μM ZnCl_2_), rinsed for 10 min in incubation buffer (1% Triton X-100, 50 mM TRIS-HCl, pH 7.5, 5 mM CaCl_2_, 1 μM ZnCl_2_) at 37 °C followed by incubation in fresh incubation buffer for 24–40 h at 37 °C. The gel was stained in staining solution (0.5% (w/v) Coomassie Blue, 10% acetic acid, 40% methanol) for 1 h, rinsed with water and incubated in destaining solution (10% acetic acid, 40% methanol) until white bands were clearly visible. 10 ng of recombinant MMP-9 (Thermofisher Scientific) was loaded as positive control. Gels were quantified using ImageJ.

#### Immunohistochemistry

Immunohistochemistry on paraformaldehyde fixed, paraffin embedded, 3 μm thick sections of mouse tumor tissues was performed manually using anti-Ki67 (1:2000), anti-CD31 (1:200) (abcam), anti-cleaved Caspase3 (1:1000), anti-phospho cMET (1:1000), anti-cMET (1:1000), anti-MMP9 (1:1000) (Cell Signaling Technologies), anti-E-cadherin (1:1000) (BD Biosciences) and anti-ILEI (1:1000) [6] primary antibodies and Lab Vision™ UltraVision™ LP Detection System (Thermo Scientific) with 3,3′-diaminobenzidine (DAB) substrate (Dako) for detection according to the manufacturer’s instruction. Cell nuclei were visualized by hematoxylin staining. Histological samples were scanned using a Pannoramic MIDI slide scanner (3D Histech) with a 40X objective. Subsequently, quantification of immunomhistochemistry was performed by the histomorphometric software package Tissue Studio® (Definiens AG). The E-cadherin membrane score was obtained by the formula: 3 x ratio of high membrane staining intensity + 2 x ratio of medium membrane staining intensity +1x ratio of low membrane staining intensity, giving a range of 1 to 3.

#### Experimental mouse xenografts

Mouse xenografts were established with subcutaneous injection of 1.7 × 10^6^ control (shCont) or ILEI KD (sh506) NCI-H1993 and NCI-H441 cells into 8–12-week old female SCID mice (*n* = 4). Injected mice were distributed into randomized cohorts and vehicle and compound treatment started as the mean tumor size reached 100 mm^3^. Crizotinib (LC Laboratories) was applied orally (50 mg/kg, dissolved in 5% DMSO, 10% ethanol and 10% Cremophor) in a 5-days treatment 2-days pause protocol. For NCI-H1993 tumors, at the time point of sacrifice of vehicle-treated mice, crizotinib treated animals were monitored for an additional 11 days without further supply of the compound. Tumors were measured regularly by a caliper and tumor volume was calculated by the formula *a* x *b*^2^ / 2 (*a* for the major and *b* for the minor tumor diameter). The tumors were dissected, and tumor mass was determined 40–50 days after injection.

#### Statistical analysis

Data are expressed as the mean ± standard error of the mean (SEM) where applicable. Data normality was checked using Shapiro-Wilk and Kolmogorov-Smirnov tests. Statistical significance was determined by unpaired two-sided Student’s *t*-test, one-way and two-way analysis of variance (ANOVA) tests and Kruskal-Wallis tests followed by Dunn’s multiple testing adjustment using Graph Prism software (version 5.0). Survival differences were calculated using log rank tests. *p* < 0.05 was considered significant. Kendall’s tau-b tests and Chi-square tests were calculated using R software (version 3.2.1). Alluvial plots were generated using Caleydo 3.0 software [18].

## Results

### Gene amplification of *FAM3C* and *MET* is tightly linked in several human carcinomas and correlates with increased gene expression and poor prognosis

**Fig. 1:**
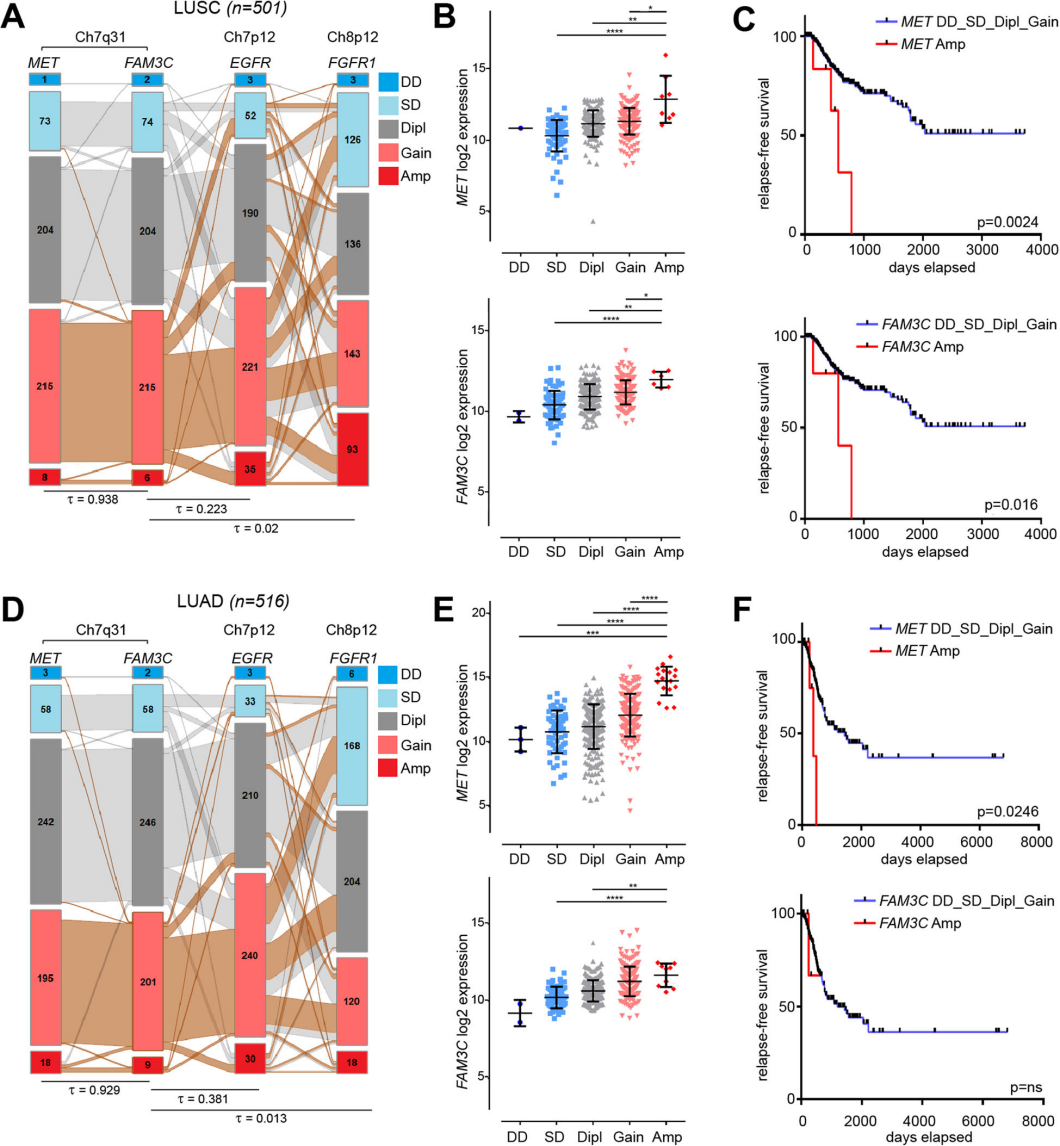
*FAM3C* and *MET* CNs are tightly linked, correlate with gene expression and survival in lung cancer. **a**, **d** Summary of *MET*, *FAM3C*, *EGFR* and *FGFR1* copy number calls extracted from lung squamous cell carcinoma (LUSC, **a**) and lung adenocarcinoma (LUAD, **d**) TCGA datasets. Genomic localization is marked above the genes. GISTIC 2.0 values were obtained from the cBio database (http://www.cbioportal.org/): − 2, deep deletion (DD); − 1, shallow deletion (SD); 0, diploid (Dipl); 1, gain; 2, amplification (Amp). Numbers within the boxes refer to the number of tumors within a group. Orange connection lines indicate tumor samples with *FAM3C* gain or amplification. Correlation was calculated using Kendall’s tau-b test. **b**, **e** mRNA expression levels of *MET* and *FAM3C* copy number calls defined in A and D. Statistical significance was determined by one-way analysis of variance (ANOVA) followed by Dunn’s multiple comparison adjustment and marked with asterisks (*p < 0.05; **p < 0.01; ***p < 0.001; ****p < 0.0001). **c**, **f** Kaplan-Meier plot on relapse-free survival of LUSCC (**c**) and LUAD (**f**) patients without (DD_SD-Dipl_Gain) and with (Amp) *FAM3C* and *MET* amplification

To determine the frequency of CN amplification of the *FAM3C* and *MET* genes, we investigated a variety of datasets of different cancer entities from the TCGA database. Connections in gene function and physical location of the two genes were uncoupled by analysis of two additional RTKs with dedicated driver functions in the progression of many malignancies: Epidermal growth factor receptor (EGFR), located on the p arm of the same chromosome and fibroblast growth factor receptor 1 (FGFR1) located on chromosome 8. Of 501 lung squamous cell carcinoma (LUSC) cases, 8 (1.9%) indicated *MET*, out of these 6 (1.4%) also *FAM3C* CN amplifications (Fig. 1a). Of 516 lung adenocarcinoma (LUAD) cases, 18 (3.5%) had *MET* and 9 (1.7%) *FAM3C* CN amplification, 8 deriving from the MET amplification group (Fig. 1d). 1480 hepatocellular carcinoma (LIHC) cases had in 11 (0.7%) and 6 (0.4%) cases with amplification for the *MET* and *FAM3C* genes, respectively, in 5 cases with a shared amplification for both, 615 colorectal adenocarcinoma (COADREAD) cases showed CN amplification in 1 case (0.2%) shared for both *MET* and *FAM3C* and 1080 breast cancer (BRCA) cases had CN amplification in 10 cases (0.9%) for both *MET* and *FAM3C*, 7 sharing amplification for both (Supplemental Fig.S1A). Correlation analyses showed that the *FAM3C* gene CNs were tightly correlated with CNs of *MET* but not with the distant *EGFR* or unlinked *FGFR1* genes in all analyzed data cohorts for LUSC (Fig. 1a), LUAD (Fig. 1d), LIHC, COADREAD, and BRCA (Supplemental Fig.S1A), indicating that co-amplification might be a consequence of chromosomal proximity.

To test if genomic amplification influenced gene expression, *MET* and *FAM3C* gene CNs were then compared to mRNA levels and relapse-free survival of LUSC and LUAD patients from the TCGA database. The analysis showed that CNs of both genes significantly correlated with gene expression levels, patients with strong *MET* and/or *FAM3C* amplification showing the highest expression of these genes (Fig. 1b and e). Patients with *MET* and/or *FAM3C* amplification also had a significantly worse survival compared to the pooled cohort of patients with deletions, normal, or slight gain in the CN of the two gene loci, albeit low case number and early loss on patient follow-up prevented a proper analysis on the survival of LUSC *FAM3C* amplified patients (Fig. 1c and f). These data indicate that the *FAM3C* and *MET* genes are frequently co-amplified in human cancers contributing to increased gene expression and poor survival.

We then tested the linkage of the two genes on genomic DNA isolated from formalin fixed paraffin-embedded tumors of 49 advanced-stage colorectal carcinoma patients by qPCR. Over 72% (24/33) of the *FAM3C-* and/or *MET*-amplified tumors showed a co-amplification of the two genes, further supporting the tight linkage of these loci (Supplemental Fig.S1B). In addition, cluster and correlation analysis of *FAM3C* amplification with available clinicopathological parameters elucidated a significant enrichment of *FAM3C* amplification in patients with extramural venous invasion (EMVI) (Supplemental Fig.S1B, C). EMVI, the spreading of cancer cells into the nearby blood vessels, is an invasive characteristic connected to worse prognosis. Although ILEI has not been linked so far to EMVI, our finding is in accordance with the described function of ILEI in inducing EMT and invasion and reflects that *FAM3C* amplification might affect gene function resulting in a clinically worse outcome. So, these results support the database analysis showing frequent co-amplification of *MET* and *FAM3C* and the likelihood of poor survival rates in patients with increased CNs of these genes.

### Increased CNs of *FAM3C* and *MET* are tightly linked and frequently present in multiple human cancer cell lines

**Fig. 2:**
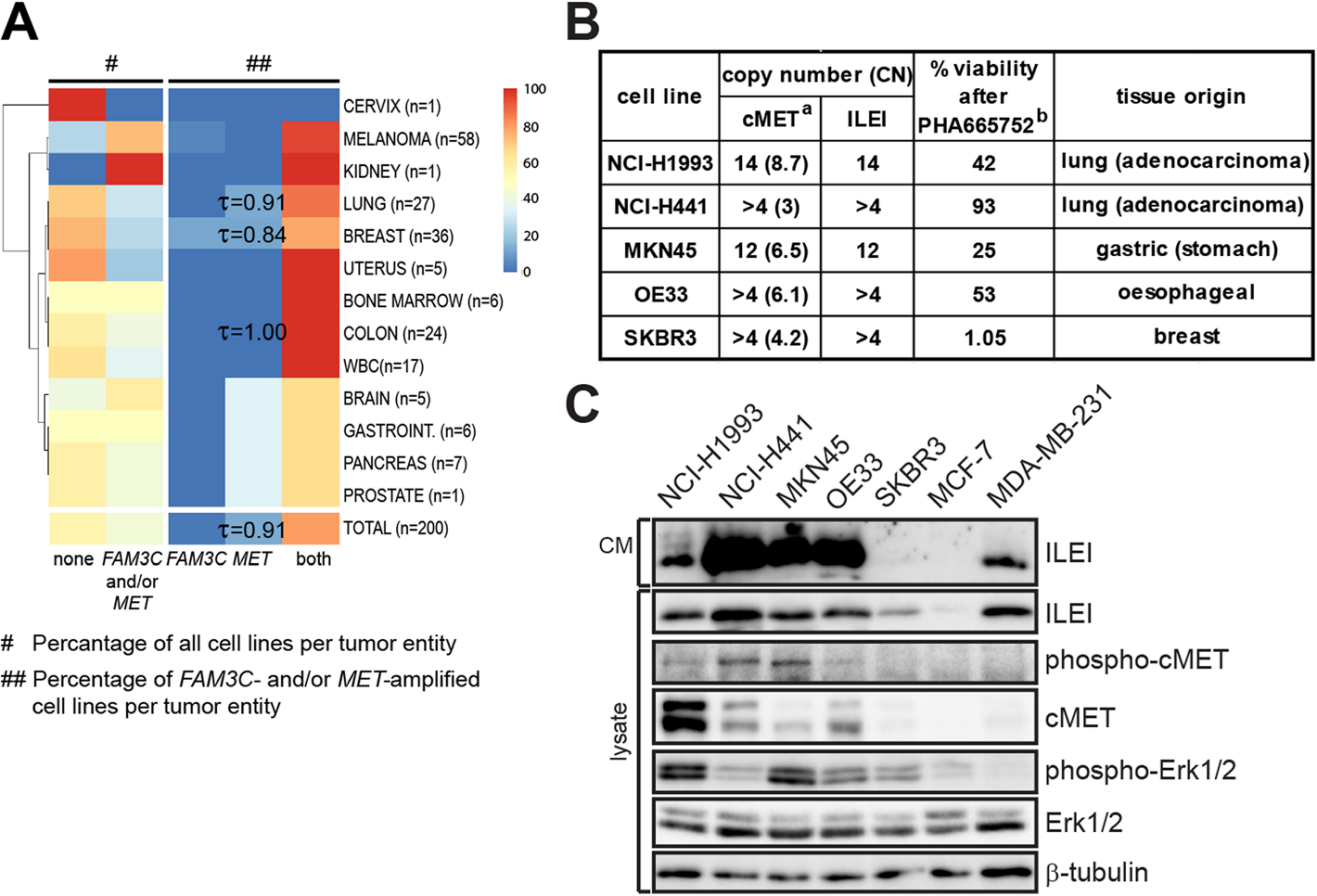
Increased *FAM3C* and *MET* CNs are frequently combined and present in human cancer cell lines. **a** Percentage of cell lines bearing increased *FAM3C* and/or *MET* gene CNs (amplification cut-off was set CN ≥ 3) determined by GeneChip® Mapping Assay in a panel of 200 cancer cell lines of diverse tissue origin. Left panel (#), percentage of cell lines with increased *FAM3C* and/or *MET* CNs; right panel (##), percentage of combined *FAM3C* and *MET* CN increase within the *FAM3C-* and/or *MET*-amplified cell lines. Number of analyzed cell lines for each tumor entity are listed on the right side. Correlation, marked for LUNG, BREAST, COLON and TOTAL, was calculated using Kendall’s tau-b test. WBC; white blood cell. **b** Short-list of five selected human tumor cell lines from panel A with increased *FAM3C* and *MET* CNs and different sensitivity to the MET inhibitor PHA665752. *MET* CNs in brackets and % viability after PHA665752 treatment are taken from [19]. **c** Western blot analysis of ILEI secretion into conditioned media (CM) and expression within the cells and MET and Erk expression and activity in above five selected cell lines. MCF-7 and MDA-MB-231 human breast cancer cell lines were used as normal *FAM3C* CN controls with low and high ILEI expression

To evaluate if our findings on *FAM3C-MET* co-amplification in human primary tumors can be recapitulated in cultured human cancer cell lines, *FAM3C* and *MET* gene CNs were determined in a panel of 200 human cancer cell lines of diverse tissue origins by microarrays and analysed using the CNAT 4.0 analysis algotrythms in the GTC analysis (Fig. 2a). Increased CN, using a cut-off of CN 3 or higher, of both genes was present in cell lines of all tumor entities at a frequency of 47% on average. The frequency varied in the different cancer types, breast and lung cancer had the lowest (25 and 30%) and melanomas the highest (76%) (Fig. 2a, left panel). Importantly, over 90% of the cell lines with an increased CN for at least one of the genes showed an increase for both loci (Fig. 2a, right panel), confirming that the amplification event of the two genes is tightly coupled and indicating that cancer cell lines representatively illustrate in vitro the *FAM3C-MET* co-amplification characteristics of primary tumors.

Of the 85 cell lines with increased CN for both *FAM3C* and *MET,* we selected five for detailed investigation (Fig. 2b). These were a pair of gastrointestinal cancer cell lines (MKN45 and OE33), a pair of lung adenocarcinoma cancer cell lines (NCI-H1993 and NCI-H441), and a breast cancer cell line (SKBR3). One cell line in each of the gastrointestinal and lung adenocarcinoma pairs had previously been described as sensitive and the other as resistant to the c-MET inhibitor PHA665752 [19]. Both these pairs of cell lines expressed high levels of ILEI and c-MET (Fig. 2c) as compared to control samples from non-metastatic MCF7 and the metastatic MDA-MB-231 human breast cancer cell line lacking *FAM3C* and *MET* amplifications but with upregulated ILEI expression as a characteristics of metastatic capacity [4]. These cell lines with high expression levels also secreted ILEI into the CM during culture. In SKBR3 cells, c-MET expression is absent despite locus amplification due to epigenetic silencing [5]. Accordingly, this cell line did not express c-MET and interestingly, though not silenced, ILEI expression was also only moderate and secretion almost absent despite increased gene CN (Fig. 2c).

### Stable ILEI knock-down does not influence proliferation capacity and sensitivity towards c-MET-inhibitor induced proliferation arrest

**Fig. 3:**
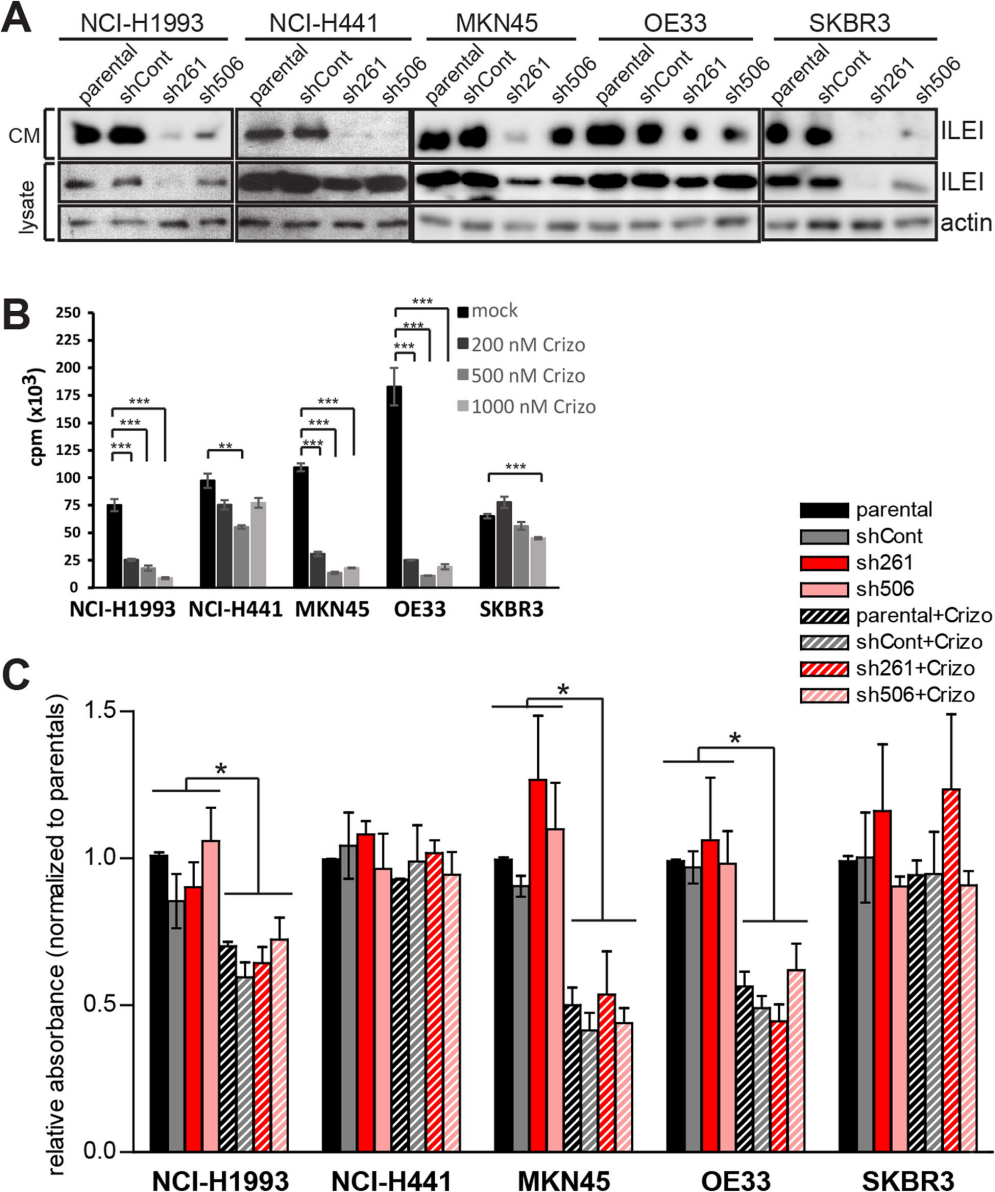
ILEI KD does not influence proliferation and sensitivity towards c-MET-inhibiton-induced arrest in *FAM3C-MET*-amplified cancer cells. **a** Western blot analysis of ILEI secretion into conditioned media (CM) and expression in the five selected cell lines (parental) and their non-targeting (shCont) and ILEI (sh261 and sh506) shRNA KD derivatives. The two independent ILEI KD lines sh261 and sh506 are named based on the starting position of the shRNA targeting site in the ILEI mRNA. **b** Proliferation behavior of the five tumor cell lines in the presence of increasing concentrations of crizotinib determined by thymidine incorporation assay. Error bars represent SEM of three independent replicates. **c** in vitro proliferation capacity of the five selected human tumor cell lines and their control and ILEI shRNA KD derivatives after 24 h vehicle or crizotinib (500 nM) treatment determined by MTT assay. Each cell line is normalized to its own parental vehicle-treated control. Error bars represent SEM of three to six independent assays. Statistical significance was determined by one-way ANOVA and marked with asterisks (*p < 0.05; **p < 0.01; ***p < 0.001)

Since there are no specific pharmacological inhibitors for ILEI available, we mimicked ILEI inhibition by RNA interference-mediated (RNAi) stable knock-down (KD) of the protein expression. Both intracellular and secreted ILEI protein levels showed an apparent reduction by two independent shRNAs in all five cell lines, which was most evident in the levels of the functionally relevant secreted form in the CM (Fig. 3a). For cMET blockade, we used crizotinib, a small-molecule tyrosine kinase inhibitor that efficiently inhibits c-MET, anaplastic lymphoma kinase 5 (ALK5) and ROS1 and is approved by the FDA for treatment of ALK-rearranged NSCLC [4]. As none of the selected cell lines including the two lung adenocarcinoma lines NCI-H1993 and NCI-H441 harbored an ALK rearrangement, inhibitor effects were expected to occur primarily due to c-MET inhibition. Since SKBR3 cells did not express c-MET despite of a *MET* and *FAM3C* amplification, they served as a control to monitor the potential influence of ALK5 and ROS1 targeting effects of crizotinib.

First, we analyzed the dose-dependency of crizotinib on the proliferation capacity of the five selected cancer cell lines. The OE33 cell line that had previously shown resistance to another small molecule c-MET inhibitor displayed sensitivity to increasing concentrations of crizotinib in proliferation capacity (Fig. 3b), comparable to the sensitive cell lines NCI-H1993 and MKN45. NCI-H441 and SKBR3 tolerated high doses of the drug without remarkable drop in their proliferation rate or viability (Fig. 3b), confirming their described resistance towards MET inhibitors [19]. ILEI KD did not influence the proliferation behavior of the selected five cell lines (Fig. 3c). Furthermore, ILEI KD also did not influence the sensitivity of these cells towards crizotinib-induced growth arrest (Fig. 3c), indicating that ILEI does not affect proliferation and does not influence c-MET-dependent regulation of proliferation. To address any concerns of the polypharmacological action of crizotinib, which inhibits other targets such as ALK5, we also investigated the action of two additional c-MET inhibitors: PHA665752 and savolitinib. The results showed similar effects on cell viability as crizotinib (Supplemental Fig.S2).

### ILEI KD impairs both c-MET-independent and c-MET-dependent invasion of cancer cells with *FAM3C* and *MET* CN gains

**Fig. 4:**
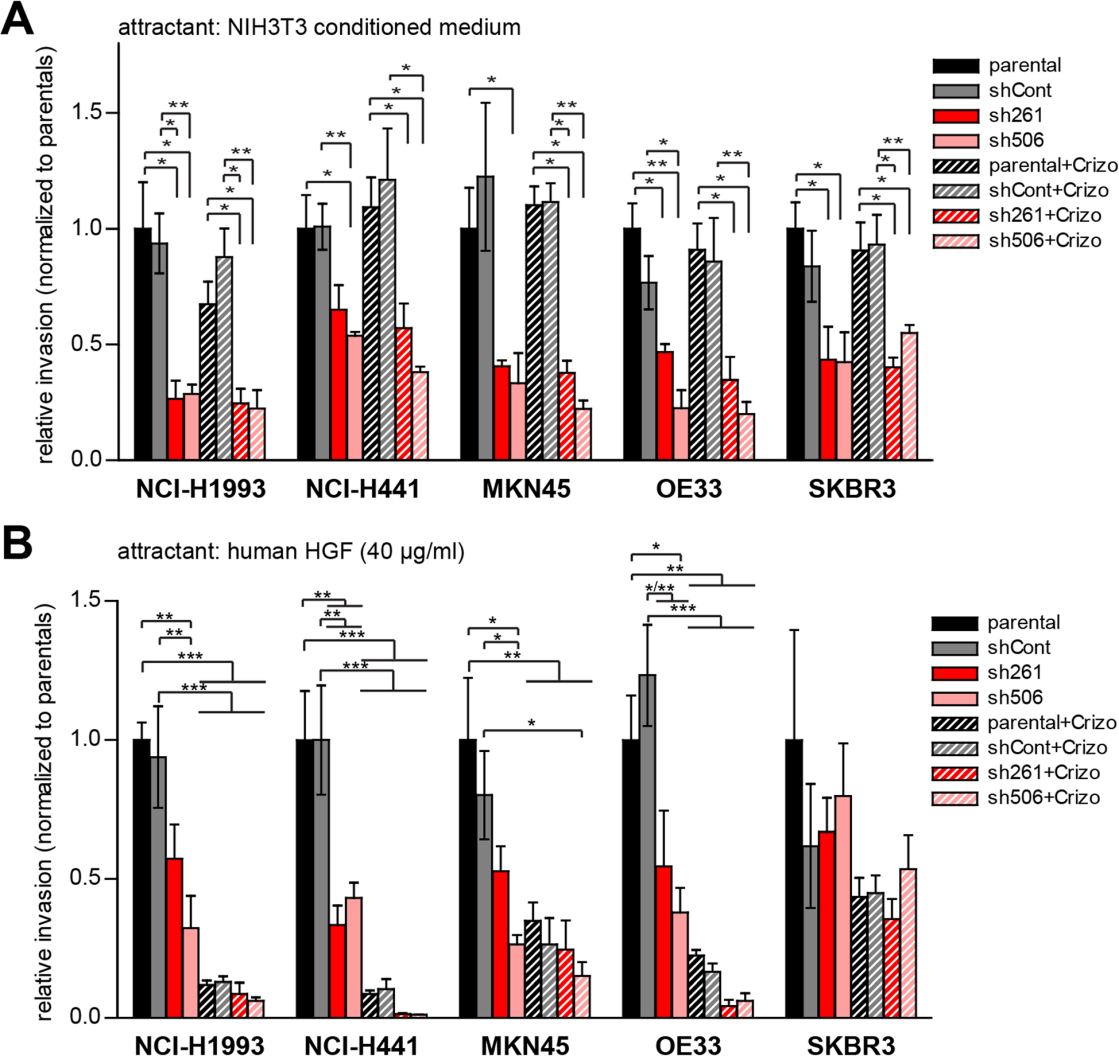
ILEI KD impairs both c-MET-independent and c-MET-dependent invasion of cancer cells with *FAM3C*-*MET* amplification. **a**, **b** Invasion capacity of the five selected tumor cell lines (parental) and of their non-targeting (shCont) and ILEI (sh261 and sh506) shRNA KD derivatives upon 24 h vehicle or crizotinib (500 nM) treatment determined by trans-well invasion assay using (**a**) conditioned medium of NIH3T3 cells or (**b**) human recombinant HGF (40 ng/ml) as chemoattractant. Each cell line is normalized to its own parental vehicle-treated control. Error bars represent SEM of three independent assays. Statistical significance was determined by one-way ANOVA and marked with asterisks (*p < 0.05; **p < 0.01; ***p < 0.001; ****p < 0.0001)

Next, we investigated invasiveness and its sensitivity to c-MET and ILEI signaling inhibition in the five selected cancer cell lines. First, we tested c-MET-independent invasive capacity in an in vitro trans-well invasion assay by using NIH3T3 CM as chemoattractant, as murine HGF produced by these cells does not cross-activate the human c-MET receptor [20, 21]. ILEI KD strongly impaired the invasiveness of all five cancer cell lines, whereas invasion capacity was not influenced by crizotinib treatment (Fig. 4a). This supported the view that ILEI signaling induces invasiveness. To test the influence of ILEI on c-MET induced invasion, the same assay was performed this time using human HGF as chemoattractant (Fig. 4b). As expected, crizotinib efficiently inhibited HGF-induced invasion in the four c-MET-expressing cell lines. Importantly, ILEI KD also significantly impaired HGF-induced invasiveness in all c-MET-expressing cells, indicating that ILEI might be a contributing factor in c-MET-driven cellular invasion. ILEI KD derivatives of NCI-H441 and OE33, even showed an increased sensitivity towards crizotinib with invasion almost completely eliminated, suggesting that ILEI depletion might have an additive inhibitory effect to crizotinib in these cells.

In summary, stable ILEI KD efficiently reduced both c-MET-dependent and c-MET-independent invasion in all tested cells.

### While ILEI does not influence c-MET signaling activity, c-MET acts on ILEI signaling activity by regulating ILEI secretion

**Fig. 5:**
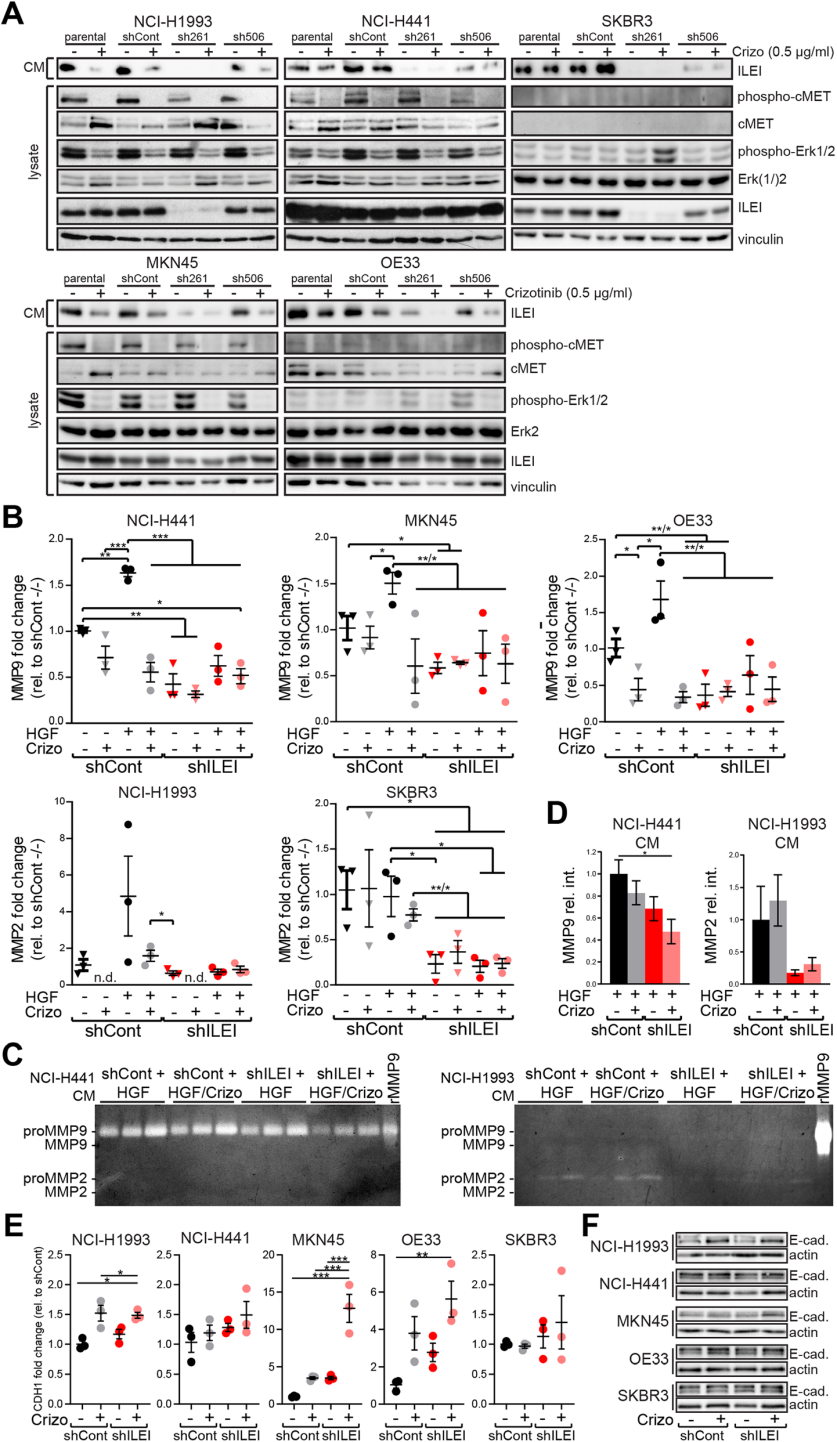
HGF-induced expression and secretion of MMPs requires ILEI, efficient ILEI secretion requires c-MET signaling. **a** Western blot analysis of ILEI secretion and expression, and c-MET and Erk activity and expression in the five selected cell lines (parental) and their control (shCont) and ILEI KD (sh261 and sh506) derivatives after crizotinib (500 nM) treatment for 24 h. **b** qPCR analysis of MMP-9 (for NCI-H441, MKN45 and OE33) and MMP-2 (for NCI-H1993 and SKBR3) mRNA expression in control (shCont) and ILEI KD (shILEI) cells after 24 h of HGF treatment (40 ng/ml) in the absence or presence of crizotinib (500 nM). Data are normalized as fold change to untreated control cells. Error bars represent SEM of three independent experiments. Statistical significance was determined by one-way ANOVA. **c** Secretion of MMP-9 and MMP-2 by control (shCont) and ILEI KD (shILEI) NCI-H441 and NCI-H1993 cells treated with HGF (40 ng/ml) for 24 h in the absence or presence of crizotinib (500 nM) determined by gelatin zymography from harvested conditioned medium. The three lanes of each treatment group represent samples of three independent assays. Recombinant pro-MMP-9 was used as assay control. **d** Quantification of the gelatin zymography gels shown in C. Relative differences in secreted MMP-9 and MMP-2 levels were determined by ImageJ analysis and normalized to HGF treatment-induced control cells. Error bars represent SEM of three independent experiments. Statistical significance was determined by Student’s t-test. **e** qPCR analysis of E-cadherin mRNA expression (CDH1) in NCI-H1993, NCI-H441, MKN45, OE33 and SKBR3 control (shCont) and ILEI KD (shILEI) cells treated or non-treated with crizotinib (500 nM) for 24 h. Data are normalized as fold change to untreated control cells. Error bars represent SEM of three independent experiments. Statistical significance was determined by one-way ANOVA and marked with asterisks (*p < 0.05; **p < 0.01). **f** Representative Western blot analysis of E-cadherin expression in the control (shCont) and ILEI KD (shILEI) derivatives of the five selected cell lines after crizotinib (500 nM) treatment for 24 h

As c-MET and ILEI interact during c-MET-dependent invasion, we tested the possibility of the two signaling pathways being linked by investigating the effect of crizotinib and ILEI KD on c-MET signaling. Crizotinib efficiently inhibited c-MET autophosphorylation in all four c-MET-expressing cell lines (Fig. 5a). Activation of Erk, an important downstream effector of c-MET, was also significantly reduced upon drug treatment in these cells, whereas it remained unaltered in SKBR3 cells, which do not express c-MET (Fig. 5a). Knock-down of ILEI did not have an influence on the expression and activation levels of c-MET and Erk (Fig. 5a). Similarly, c-MET inhibition did not influence ILEI expression levels in the tested cell lines (Fig. 5a), indicating that c-MET and ILEI expression is not cross-regulated. Importantly, however, crizotinib decreased the secretion of ILEI in all c-Met expressing cell lines, but not in SKBR3 cells (Fig. 5a). Similar results were also found with the c-MET specific inhibitor savolitinib (Supplemental Fig.S3A). This suggests that c-MET may positively regulate ILEI secretion.

### Elevated expression and secretion of MMPs upon c-MET activation depends on ILEI and the two pathways cooperate in E-cadherin repression

To uncover potential mechanisms by which c-MET and ILEI might cooperate to increase invasiveness, we next investigated markers of invasion. MMPs remodel the extracellular matrix (ECM) and are activated during invasion to ease the movement of cancer cells [22]. So, we explored the mRNA expression levels of two prominent MMPs, MMP-2 and 9 in response to HGF in the five cell lines. Of note, none of the five cells showed expression of both of these MMPs; NCI-H441, MKN45 and OE33 expressed only MMP-9, whereas NCI-H1993 and SKBR3 cells only MMP-2. Firstly, ILEI KD lead to decreased expression of MMP-9 or MMP-2 in all five cell lines, confirming its role in invasion and MMP expression (Fig. 5b) and [17]. Secondly, in NCI-H441, MKN45, and OE33 cells HGF induced expression of MMP-9 mRNA and this expression was inhibited by crizotinib supporting the view that c-MET-dependent invasion activates MMP-9 (Fig. 5b) and [23, 24]. Even more importantly, however, in ILEI KD derivatives of these cells HGF treatment was not able to elevate MMP-9 mRNA expression (Fig. 5b). This result suggests that c-MET-mediated increased expression of MMP-9 mRNA during invasion is dependent on ILEI. A similar pattern was seen in NCI-H1993 cells for MMP-2 mRNA expression, but not in SKBR3 cells, where c-MET is not expressed and hence, HGF did not increase MMP-2 mRNA levels (Fig. 5b). As MMPs act on ECM, their activity is dependent upon secretion, so we analyzed the effect of c-MET and ILEI signaling inhibition on MMP secretion. For easier detection of inhibitory effects, high baseline secretion was ensured by HGF trigger. The secretion of MMP-9 from NCI-H441 cells decreased slightly with crizotinib treatment or ILEI KD, and the combination of the two lead to a significant reduction (Figs. 5c, d). In NCI-H1993 cells ILEI KD was sufficient for remarkable reduction of MMP-2 secretion (Fig. 5c, d). The specificity of these results to c-MET inhibition was also tested with savolitinib with similar results (Supplemental Fig.S3b). Overall, these results suggest that both c-MET and ILEI contribute to efficient secretion of MMPs in a cooperative and partially complimentary manner, c-Met most probably acting indirectly, via regulating ILEI secretion.

Another important marker of invasion and EMT status is the loss or reduction of the cell adhesion molecule E-cadherin [25]. Therefore, we checked potential changes in the levels of E-cadherin mRNA (CDH1) and protein upon crizotinib treatment and ILEI KD in each of the cell lines used in this study, and found high variance according to the cell type (Fig. 5e, f). On the one hand, in the crizotinib-resistant NCI-H441 and SKBR3 cells no significant differences in CDH1 mRNA expression levels were observed (Fig. 5e). On the other hand, NCI-H1993, MKN45 and OE33 cell lines showed a significant c-MET and ILEI mediated regulation of CDH1 transcription. All three cell lines showed increase in CDH1 mRNA levels in ILEI KD derived cells or with crizotinib, and combination of these conditions resulted in more stable or superior effects. Cells tested with savolitinib showed similar results (Supplemental Fig.S3c). E-cadherin protein levels showed a similar trend of differences as of transcription (Fig. 5f). This once again suggests a cooperation between c-MET and ILEI in the regulation of E-cadherin transcription, however, these data also point out that cancer cells show very different sensitivity towards this regulation.

### Combined ILEI KD and crizotinib treatment significantly reduced the outgrowth of NCI-H441 and NCI-H1993 tumor xenografts

**Fig. 6:**
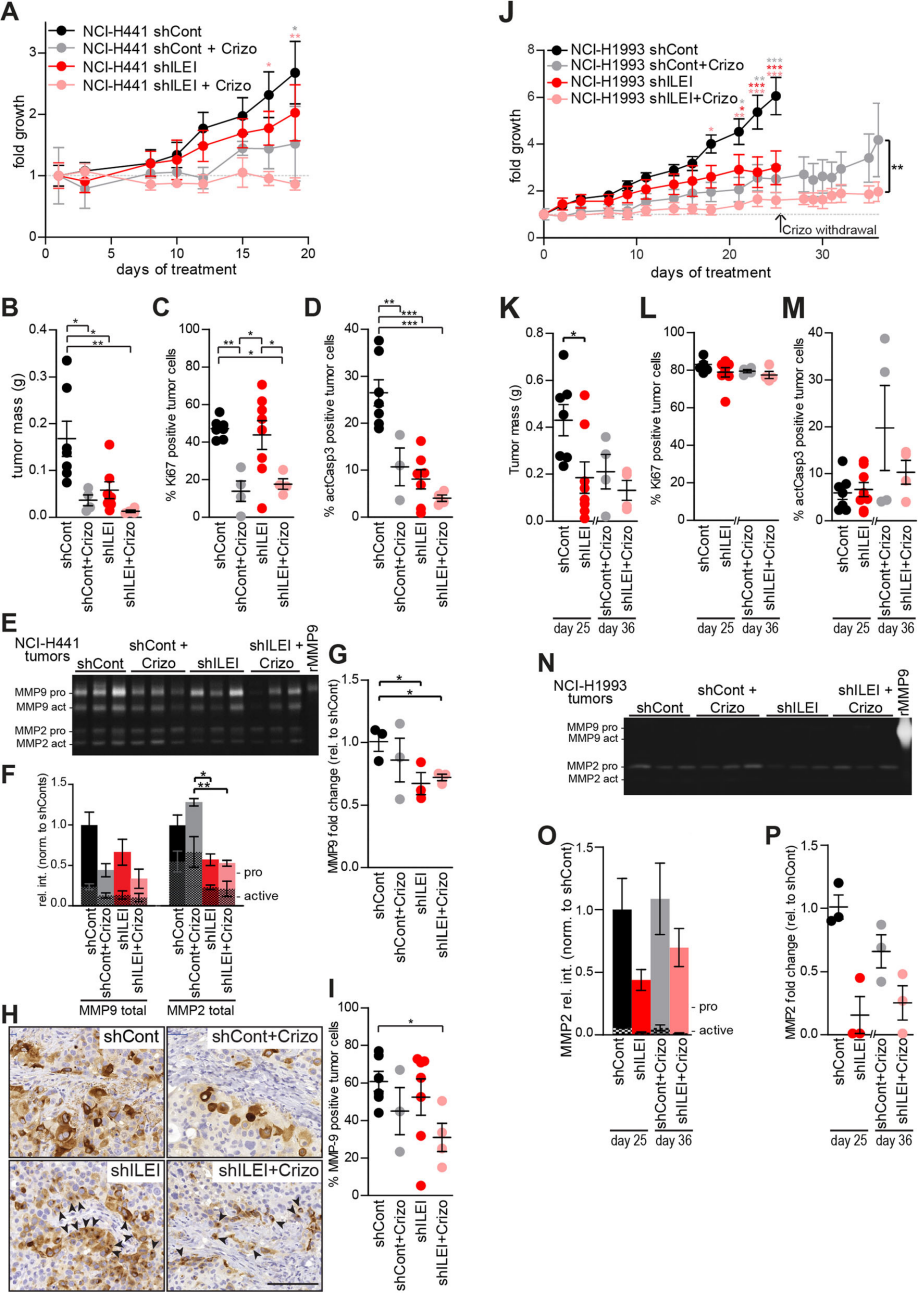
Combined ILEI KD and crizotinib treatment reduces tumor growth by inhibiting proliferation and MMP expression. **a**, **j** Fold growth ± SEM of NCI-H441 (**a**) and NCI-H1993 (**j**) control (shCont) and ILEI KD (shILEI) tumors upon vehicle or crizotinib (Crizo) treatment normalized to size at treatment start. Crizotinib-treated NCI-H1993 tumors were allowed to grow for an additional 11 days after treatment termination. **b**, **k** Tumor masses ± SEM of NCI-H441 (**b**) and of NCI-H1993 (**k**) shCont and shILEI tumors of vehicle or Crizo-treated mice. **c**, **l** Percentage of Ki67 positive tumor cells ± SEM of NCI-H441 (**c**) and NCI-H1993 (**l**) shCont and shILEI tumors of vehicle or Crizo-treated mice. **d**, **m** Percentage of activated caspase 3 (actCasp3) positive tumor cells ± SEM of NCI-H441 (**d**) and NCI-H1993 (**m**) shCont and shILEI tumors of vehicle or crizotinib-treated mice. **e**, **n** Gelatin zymography of protein extracts of NCI-H441 (**e**) and NCI-H1993 (**n**) shCont and shILEI tumors of vehicle- or crizotinib-treated mice (*n* = 3 pro group). Recombinant pro-MMP9, assay control. **f**, **o** Quantification of gels of panel **e** (**f**) and n (**o**). Pro- (filled color) and activated (patterned color) MMP-9 and MMP-2 levels ± SEM were normalized to respective total MMP levels of vehicle-treated control tumors. Statistics compares total MMP-9 and MMP-2 levels. **g**, **p** mRNA expression levels of MMP9 in NCI-H441 (**g**) and of MMP2 in NCI-H1993 (**p**) shCont and shILEI tumors of vehicle- or crizotinib-treated mice (*n* = 3 pro group). Expression was normalized to GAPDH and shown as fold change ± SEM over vehicle treated control tumors. **h** Representative images of MMP-9 IHC on NCI-H441 shCont and shILEI tumor sections of vehicle or crizotinib-treated mice. Arrowheads mark intracellular granular MMP-9 localization. Scale bar, 100 μm. **i** Percentage of MMP-9 positive tumor cells ± SEM in NCI-H441 shCont and shILEI tumors of vehicle or crizotinib-treated mice. Statistical significance was determined by two-way ANOVA (**a**, **j**), Student’s t-test (**a**, **j**) and one-way ANOVA (**b**, **c**, **d**, **f**, **g**, **i**, **k**, **l**, **m**, **o**, **p**) and is marked with asterisks (*p < 0.05; **p < 0.01; ***p < 0.001)

To assess the in vivo relevance of the above findings, we next investigated the growth of tumor xenografts induced by NCI-H441 and NCI-H1993 cells and their ILEI KD derivatives in the presence and absence of crizotinib treatment in a mouse model. The original rationale was to compare the effect of ILEI KD on the growth capacity of crizotinib-sensitive vs crizotinib resistant tumors. Because we were expecting a very low response of the NCI-H441 cell line towards crizotinib we did not plan a withdrawal step for that cell line, while the NCI-H1993 cell induced xenografts were evaluated for crizotinib withdrawal for an additional 11 days. However, in accordance with some earlier findings [26], we observed that inhibition of c-MET with crizotinib reduced the growth and tumor mass of both of the xenografts (Fig. 6a, b, j, k), indicating that crizotinib was able to counteract the resistance of NCI-H441 cells most probably via non-cell intrinsic mechanisms not addressed here. Importantly for this study, ILEI KD also slowed the growth of both xenografts. Tumor growth was most efficiently reduced when ILEI KD was combined with crizotinib (Fig. 6a, j). The tumor mass was significantly decreased in cells with ILEI KD compared to those with ILEI expression and was lowest in cells with ILEI KD and crizotinib combined (Fig. 6b, k). Immunohistochemistry for ILEI (Supplemental Fig. S4a, g), c-MET (Supplemental Fig. S4b, h), and phospho-cMET (Supplemental Fig. S4c, i) confirmed significant ILEI KD, unaltered c-MET expression upon ILEI KD and inhibitor treatment, as well as efficient inhibition of c-MET activation by crizotinib, respectively. The latter was also quantified as percentage of phospho-c-MET positive tumor cells (Supplemental Fig. S4d, j). It is notable that decrease in c-MET activation was no more evident in the NCI-H1993 derived tumors due to crizotinib withdrawal over the last 11 days of the experiment (Supplemental Fig. S4j).

To investigate the reasons for smaller tumors resulting from crizotinib treatment and ILEI KD we investigated the proliferation and apoptosis of the tumor cells by quantifying the percentage of Ki67 and activated Caspase3 positive cells on tissue sections, respectively. In NCI-H441 xenografts, crizotinib treatment significantly reduced the proliferation of cells, but ILEI KD alone did not influence this parameter (Fig. 6c) [26]. So, this result supported the cell-based assays and showed that the cells were behaving in a similar manner in vivo to the in vitro analysis. In NCI-H1993 xenografts, the graph shows the recovery of the tumor cells after crizotinib treatment was halted on day 25 and observed for an additional 11 days. This indicated that after a period of drug withdrawal, proliferation was apparently no longer affected by the previous crizotinib treatment (Fig. 6l). Apoptosis was also decreased in NCI-H441 xenografts treated with crizotinib and in ILEI KD derived cells (Fig. 6d). In NCI-H1993 xenografts that had a withdrawal period from the crizotinib treatment phase there was no apparent significant difference in apoptosis with crizotinib or ILEI KD (Fig. 6m). So, the smaller tumors are likely to be due to the decreased proliferation upon crizotinib treatment rather than an increase in apoptotic cell death. A higher level of apoptosis in larger tumors without c-MET inhibition or ILEI KD may be indicative of the fast turnover of cells in these rapidly proliferating tumors that was also manifested in a highly ulcerated appearance.

We also addressed if c-MET and ILEI had a consequence on tumor vascularization by determining blood vessel density and size on CD31 immunostained tumor sections. The vessel density remained constant between tumors (Supplemental Fig. S4e, k), and though NCI-H441 tumors showed decreased vessel size upon crizotinib treatment, it was less evident in the tumors with ILEI KD and not evident in any of the NCI-H1993 tumors (Supplemental Fig. S4f, l). These data indicate that decreased tumor size upon combined ILEI KD and c-MET inhibition is not primarily due to a switch in vascularization capabilities.

### Combined ILEI KD and crizotinib treatment decreased MMP expression in NCI-H441 and NCI-H1993 tumor xenografts

To investigate whether the relationship between MMPs, c-MET, and ILEI seen in the cell lines was also evident in vivo, the expression of MMP-9 and MMP-2 from the tumors was investigated. In line with the results from gelatin zymography, the expression of MMP-9 protein in NCI-H441 tumors decreased slightly with crizotinib inhibition and even more when the cells also had ILEI KD, while MMP-2 expression, that became detectable only at in vivo conditions, was significantly decreased in ILEI KD tumors (Fig. 6e, f). At mRNA level, there was a significant decrease of MMP-9 in tumors from ILEI KD cells (Fig. 6g) and quantification of MMP-9 immunohistochemistry in tumor sections showed a similar trend with a significant difference between the shCont tumors and those with ILEI KD and crizotinib in combination (Fig. 6h, i). NCI-H1993 tumors also expressed lower levels of MMP-2 protein upon ILEI KD (Fig. 6n, o) and this result was supported at the mRNA level, though without significance (Fig. 6p). Overall, these results show that both c-MET and ILEI cooperate for efficient MMP expression during growth of tumor xenografts and support the results from the cell-based assays.

### Combined ILEI KD and crizotinib treatment increased E-cadherin membrane localization

**Fig. 7:**
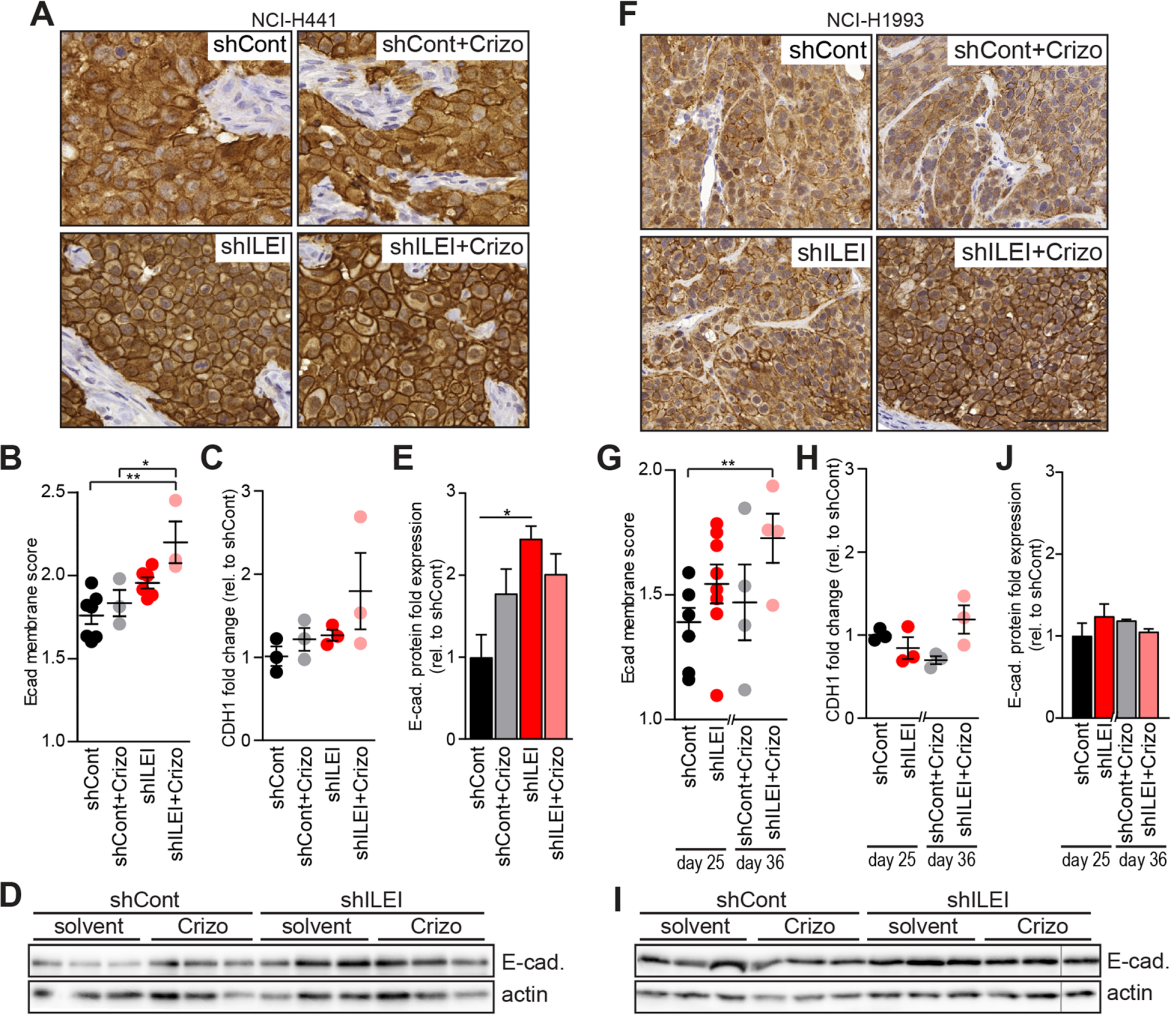
Combined ILEI KD and crizotinib treatment increases E-cadherin membrane localization in tumor xenografts. **a**, **f** Representative images of E-cadherin IHC on NCI-H441 (**a**) and NCI-H1993 (**e**) shCont and shILEI tumor sections of vehicle or crizotinib-treated mice. Scale bar, 100 μm. **b**, **g** E-cadherin membrane score of NCI-H441 (**b**) and NCI-H1993 (**f**) shCont and shILEI tumors of vehicle- or crizotinib-treated mice. Error bars represent SEM. Statistical significance was determined by one-way ANOVA and marked with asterisks (*p < 0.05; **p < 0.01). **c**, **h** qPCR analysis of E-cadherin mRNA expression (CDH1) in NCI-H441 (**c**) and NCI-H1993 (**g**) shCont and shILEI tumors of vehicle- or crizotinib-treated mice (*n* = 3 pro group). Relative expression was normalized to GAPDH and shown as fold change to vehicle treated control tumors. Error bars represent SEM. Statistical significance was determined by one-way ANOVA and marked with asterisks (*p < 0.05). **d**, **i** Western blot analysis of E-cadherin protein expression in NCI-H441 (**d**) and NCI-H1993 (**h**) shCont and shILEI tumors of vehicle- or crizotinib-treated mice (*n* = 3 pro group). **e**, **j** Quantification of E-cadherin protein expression from the Western blot analyses shown in panels **d** and **i**. Expression was normalized to actin loading control and shown as fold expression relative to vehicle treated shCont tumors. Error bars represent SEM. Statistical significance was determined by one-way ANOVA and marked with asterisks (*p < 0.05)

To further compare tumor invasiveness and EMT status, E-cadherin-mediated cell-cell adhesion was investigated. In both, NCI-H441 and NCI-H1993 xenografts immunohistochemistry of tumor sections showed a slight increase of E-cadherin at the membranes of tumors treated with crizotinib and those derived from ILEI KD cells, and this became significant when they were in combination (Fig. 7a, b, f, g). Similar to their in vitro behavior, none of the two xenografts showed regulation of E-cadherin expression at the mRNA level upon different conditions (Fig. 7c, h), nor E-cadherin levels of tumor protein extracts showed a uniform trend of regulation (Fig. 7d, e, i, j). Therefore, these results suggest that ILEI and c-MET mainly cooperate to reduce E-cadherin protein localization at the membrane to decrease cell-cell adhesion and increase the potential for invasion.

## Discussion

**Fig. 8:**
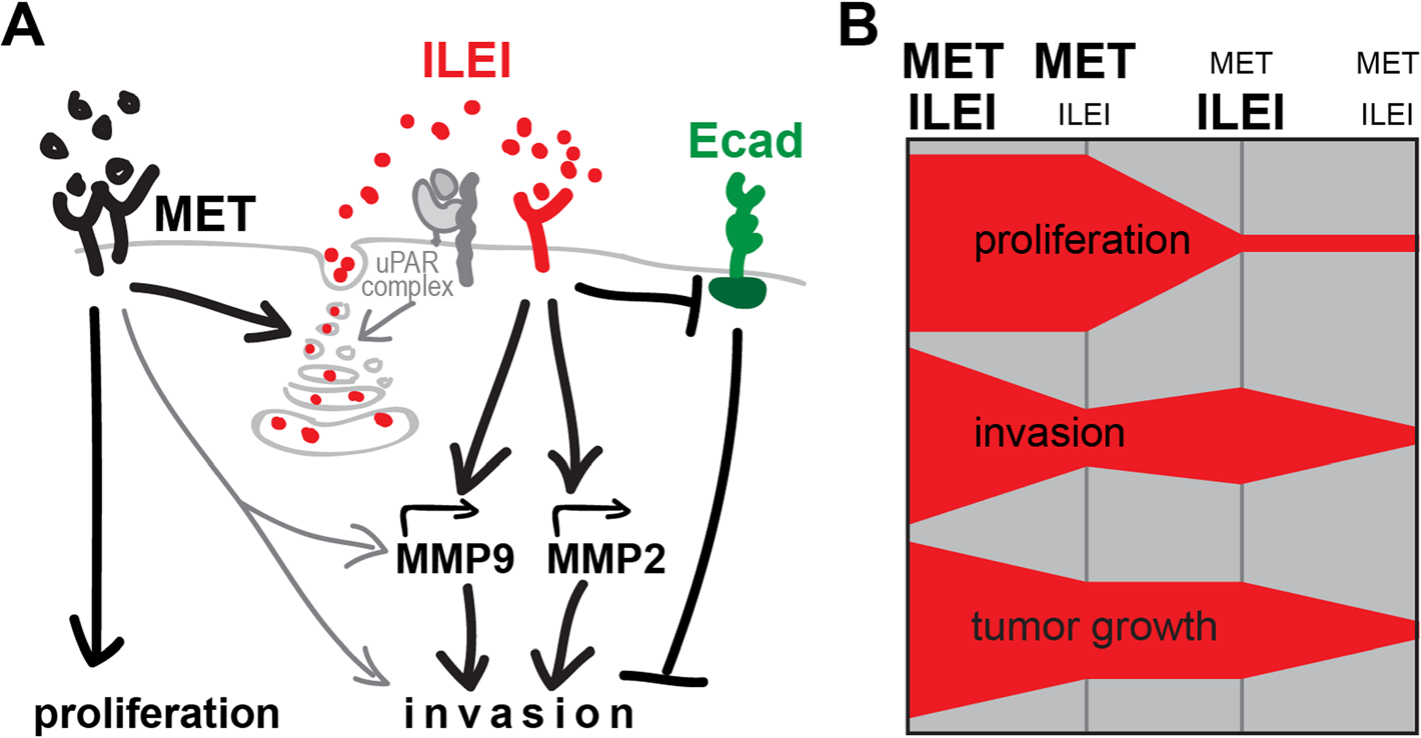
Model of ILEI and c-MET signaling interplay on invasive tumor growth. **a** Molecular interplay of ILEI and c-MET signaling on invasion. **b** The influence of c-MET and ILEI signaling levels on the net outcome of tumor proliferation, invasion and overall tumor growth

The aim of this study was to investigate whether the *FAM3C* CN contributes to elevated ILEI expression in cancer and its potential relationship to *MET*. The results show a close correlation between *FAM3C* and *MET* CNs and that cancers with high CN had higher gene expression levels of both ILEI and c-MET, which was also related to poorer outcome. Investigation of the mechanisms involved suggests that there is a cooperation between ILEI and c-MET signaling during cancer invasion as summarized in the model in Fig. 8a. During c-MET-dependent invasion, as seen in some previous studies, MMP secretion was increased [23, 24] and E-cadherin levels at the cell membranes were decreased [27, 28]. This study showed that both these processes were supported by ILEI expression and that c-MET also increased the secretion of ILEI. The secretion of active ILEI requires mobilizing its intracellular protein pool in a urokinase-type plasminogen activator receptor (uPAR)-dependent manner [13]. So, we suggest that i) c-MET might be involved in that process and ii) regulatory functions of c-Met on invasion might work indirectly via regulating ILEI secretion and thus, ILEI signaling activity. c-MET signaling was not cross-regulated by ILEI and ILEI did not have an influence on c-MET-dependent proliferation in cancer cell lines, showing that the interplay between these two signaling pathways on proliferation, invasion, and overall tumor growth acts rather in a complimentary manner as shown in Fig. 8b.

Although the *MET* gene undergoes many different types of mutation to become oncogenic, amplification of the *MET* locus has been reported in a variety of human cancers [4]. Amplified *MET* CN has been shown to negatively influence patient survival in a lot of cancer types including esophageal squamous cell carcinoma [29], NSCLC [30], clear-cell renal cell carcinoma [31], and ovarian carcinoma [32]. However, this association with poor prognosis is not always evident with high c-MET protein expression [32, 33], highlighting the complexities of the *MET* amplicon’s involvement in cancer. This observation also suggests that additional genes of the amplified region might contribute to poor patient survival. The focus of this study concentrated on one of *MET*’s closest neighbors, *FAM3C*. For its gene product, ILEI, high levels of protein expression correlated with poor prognosis in colorectal cancer [14]. We found in this study that *FAM3C* frequently co-amplified with *MET* and patients with *MET* and *FAM3C* amplification had poor prognosis. The high efficiency of the combined inhibition of c-MET and ILEI function on the inhibition of invasion and tumor growth of cancer cells bearing *MET* and *FAM3C* amplifications found in this study justifies the relevance of this co-amplification on clinical outcomes. Other neighboring genes in close proximity with possible co-amplification, e.g. *Wnt* family members and *B-Raf*, might have additional modulatory effects on c-MET and/or ILEI action and future studies of them will be interesting to fully resolve all functionally important players of this amplification hotspot in cancer.

A previous study about the variety of *MET* mutations in cancer used database analysis of 14,466 cancer cases and identified 186 cases with *MET* CN amplification [34]. This is around 1%, similar to the TCGA database analysis in our study which showed CN amplification rates of 1.9% (8 in 501) for LUSC cases, 3.5% (18 in 516) for LUAD cases, 0.7% (11 of 1480) for LIHC cases, 0.2% (1 of 615) for COADREAD cases, and 0.9% (10 in 1080) for BRCA cases, with a total rate of 1.1% (48 in 4192). For further comparison, increased *MET* gene CN measured by fluorescence in situ hybridization was found in 1 to 4% of tumors from NSCLCs [30], 8.5% of lung sarcomatoid carcinomas [35], and 4.2% of colorectal cancer tissue samples [36]. However, these values are lower than the 47% rate in cancer cell lines and 67% in colon carcinoma patient samples that had CN amplification of *MET* and *FAM3C*. The main reason for the differences observed between database, cell lines and patient samples are different cut-off points. We considered a CN of 3 or higher to be amplification in qPCR and gene chip analyses of this study, while databases use higher cut-off points and a CN of 3 would be recorded as a gain rather than amplification. When the higher numbers of cases with gains in CN were added to the amplified CNs for the TCGA analysis the overall rate of CN amplification increased to nearly 30% of the total cases, which is much closer to the rates for the cell lines and COAD patients. The differences in CN frequencies that remain between the different samples might result from various factors. As c-MET activation leads to increased proliferation and growth of cancer cells, it is possible that this type of growth factor receptor gene amplification will be of benefit during selection of stable cancer cell lines. Indicating that, cancer cell lines might be more likely to gain CN amplifications. Furthermore, the 49 colorectal carcinoma cases in this study were all advanced stage cancer patients at stage T3 and T4, with 55% already displaying lymph node and/or distant metastasis, which as our data suggests have invasion and poorer prognosis, that might result an additional enrichment of *MET* and *FAM3C* CN. Our results are also in agreement with the conclusion of the above mentioned database analysis that *MET* CNs in general show a wide variation among cancer types, albeit relative frequencies for different tumor entities show differencies between the studies.

All these data show that *MET* amplification has broad relevance in many cancers and our study indicates that *FAM3C* co-amplification may play a comparably important role in all these different cancer types. As the alternative name of c-MET indicates, Hepatocyte Growth Factor Receptor (HGFR) has an important role in liver development and regeneration [37], thus predestinating a pivotal oncogenic driver function of the gene in liver cancer. Indeed, aberrant c-MET function, including gene amplification is frequent in HCC [38]. However, the above statistics show that it is not less frequent in other epithelial cancer types, and *MET* amplification is one of the frequent aquired alterations (5–20% of patients) upon resistance towards EGFR tyrosine kinase inhibitor (TKI) therapies in lung cancer [39]. Thus, our mechanistic studies on NSCLC cell lines with *MET* amplification are of direct clinical relevance to many cancer types.

c-MET signaling involves many different processes and significant crosstalks with other signaling pathways. For example, there is an interaction between c-MET signaling and the vascular endothelial growth factor (VEGF) and VEGF receptor (VEGFR) pathways [40]. While interactions between c-MET and human epidermal growth factor receptor (HER) family members allow tumor progression and treatment resistance, and cooperative signaling between c-MET and HER2 might be a mechanism by which c-MET promotes cancer progression [41]. Interestingly, in breast cancer cells the ILEI-uPAR score, which is indicative of the potential for active secreted ILEI, was shown to be significantly correlated with the HER2 status of the tumor cells [13] suggesting that these three signaling pathways may cooperate to increase invasiveness. In addition, uPAR-bound activated uPA is required for the proteolytic maturation of the c-MET ligand HGF [4]. At the same time, active uPA is needed for the activation of Plasminogen, the protease responsible for the maturation of ILEI, and activation of the uPA-uPAR system has also a key role in triggering ILEI secretion [13]. This shared use of the same proteolytic cascade by both signaling pathways for activation also indicates a strong positive regulatory connection. Because of the importance of c-MET in many cancer types, inhibitors of c-MET are in clinical trials as cancer treatment, but a significant percentage of tumors acquire resistance to these treatments [4, 19]. This may in part be due to the ability of c-MET to crosstalk and interact with alternative RTKs such as VEGFR and HER2 [40, 41]. Combination treatments may help address this problem and the results of this study suggest that ILEI might be a potential target for these treatments as highlighted by the xenograft experiments that showed tumor growth was mostly inhibited by combined crizotinib and ILEI KD.

ILEI is less well understood than c-MET, but its role in cancer progression is starting to emerge. ILEI translation appears to be stimulated by transforming growth factor beta (TGF-β) and silenced by heterogeneous nuclear ribonucleoprotein E1 (hnRNP E1). Part of the signaling pathway, at least in breast cancer cells, involves the leukemia inhibitory factor receptor (LIFR) and signal transducer and activator of transcription 3 (STAT3) signaling [42]. The active form of ILEI requires proteolysis and is self-dimerized [13, 17, 43]. However, it was unclear so far whether over-expression of ILEI could result from CN amplification until this study.

Once we had established that *MET* and *FAM3C* were often co-amplified and this led to combined overexpression of c-MET and ILEI, it was important to investigate whether they interact during cancer progression. For this, we used five cancer cell lines that had shown co-amplified *MET* and *FAM3C*. For c-MET inhibition, we used crizotinib and ILEI KD was used to mimic ILEI inhibition. Somewhat different to the published data on the MET inhibitor PHA665752 [19], OE33 cells showed high sensitivity to increasing concentrations of crizotinib in proliferation capacity and were also sensitive to both PHA665752 and savolitinib in this study. The other cell lines showed in vitro sensitivity (NCI-H1993 and MKN45) or resistance (NCI-H441 and SKBR3) towards crizotinib as expected from previous publication. ILEI KD did not influence the proliferation of the cell lines suggesting that ILEI is not involved in proliferation of cancer cells or c-MET-regulated proliferation. However, ILEI KD strongly impaired the invasiveness of all five cancer cell lines. This was in accordance with earlier findings [6] and further supported that view that ILEI signaling induces invasiveness in different types of cancer [44]. Importantly, the impairment of invasiveness by ILEI KD on HGF-induced, hence c-MET-dependent invasion suggests that c-MET driven invasion depends on ILEI. In addition, the importance of ILEI in c-MET-independent invasion suggests that ILEI has a broad influence on invasion and does not rely exclusively on c-MET secretion trigger.

In terms of invasion, an important step is ECM degradation which allows tumor dissemination. MMPs are implicated in this process because they mediate the constant remodeling of the ECM [22]. Our results suggested that ILEI was required for HGF-induced expression and secretion of MMP-9 and MMP-2 through c-MET signaling. Although the expression levels of ILEI were not influenced by crizotinib treatment and ILEI did not affect activation of c-MET signaling, there was an apparent decrease in ILEI secretion upon c-MET inhibition. This suggests c-MET may regulate MMPs indirectly via ILEI secretion. So, the independent c-MET and ILEI processes show a vital interplay and cooperation to support invasiveness.

The results of the cell-based studies were then further investigated in xenografts in mice. Our expectation was that the growth of crizotinib-sensitive tumors would be slower than for crizotinib resistant tumors when c-MET was inbitited. However, crizotinib reduced the growth and tumor mass of xenografts induced by both sensitive (NCI-H1993) and resistant (NCI-H441) cell types. This was is in accordance with some earlier findings [26] and suggested that crizotinib was able to counteract the resistence of NCI-H441 cells to inhibit c-MET, most probably via non-cell intrinsic mechanisms that will require further investigation. This result meant that we then focused on ILEI and found that both cell types showed reduced growth of tumor xenografts from ILEI KD and a superior reduction with crizotinib treatment in combination supporting the interplay between the two signaling pathways. Combination of ILEI KD and crizotinib also resulted in lower expression of MMPs in a similar way to the cell-based studies. MMP-2 and MMP-9 can degrade components of the ECM such as type IV collagen to release tension and allow growth and invasion of tumors and as such are implicated in the late stages of cancer [45]. During EMT cell-cell junctions begin to disassemble [9]. The best characterized alteration at this point involves the loss of E-cadherin, a key cell-to-cell adhesion molecule [25]. E-cadherin helps to assemble and maintain epithelial cell sheets through adherence junctions. Therefore, increased expression of E-cadherin acts as an antagonist of invasion and metastasis [46]. When E-cadherin was investigated in the xenograft tumors, combined crizotinib and ILEI KD significantly increased E-cadherin at the membranes. This was less obvious at the mRNA and protein levels. Overall, our study showed high variance among tumor cells how E-cadherin levels were regulated, indicating that it is a highly dynamic process with strong control at the transcription level, but also via internalization or altered stability in cancer cells [47]. Also, in some cancers, such as hepatocellular carcinoma, E-cadherin protein accumulation is prevented by mRNA retention in the nucleus [48].

## Conclusions

The results of this study show that amplification of *FAM3C* CN can contribute to increase the level of ILEI expression in a wide range of cancer types. There was a close correlation between *FAM3C* and *MET* CNs in cancer patients and those with high CNs had poorer outcomes. Investigation of the mechanisms involved showed interplay between the two separate ILEI and c-MET signaling pathways during cancer invasion, suggesting *MET* amplifications are in reality *MET-FAM3C* co-amplifications with tight functional co-operation. In vivo investigation showed that ILEI knock-down and c-MET-inhibition in combination significantly reduced the invasive outgrowth of lung tumor xenografts in mice, apparently by inhibiting proliferation, MMP expression and E-cadherin membrane localization. Therefore, including ILEI as a target in the therapy of cMET-amplified human carcinomas may be an effective approach for the future.

## Supplementary Information

**Additional file 1: Figure S1.** (related to Fig. 1.). *FAM3C* and *MET* gene copy numbers are tightly linked in human hepatocellular, colorectal and breast cancers with *FAM3C* amplification correlating with venous invasion in colorectal cancer patients. **a** Summary of *MET*, *FAM3C*, *EGFR* and *FGFR1* copy number calls extracted from hepatocellular carcinoma LIHC (left panel), colorectal adenocarcinoma COADREAD (mid panel) and breast carcinoma BRCA (right panel) TCGA datasets. Genomic localization is marked above the genes. GISTIC 2.0 values are shown: − 2, deep deletion (DD); − 1, shallow deletion (SD); 0, diploid (Dipl); 1, gain; 2, amplification (Amp). Numbers within the boxes refer to the number of tumors within a group. Orange connection lines indicate tumor samples with *FAM3C* gain or amplification. Correlation was calculated using Kendall’s tau-b test. **b** Heatmap with hierarchical clustering of *FAM3C* and *MET* gene amplification determined by Taqman qPCR and of extramural venous invasion (EMVI), tumor stage (pT), lymph node status (pN) and metastasis status (pM) clinicopathological parameters extracted from 49 primary colon carcinoma patients. Calculated copy numbers of 3 or higher were considered as gene amplification. Correlation was calculated using Kendall’s tau-b test. **c** Correlation of *FAM3C* gene amplification status with established clinicopathological parameters of the 49 human colorectal cancer patients shown in panel b. The number of patients (n) and their relative frequencies (in %; numbers in parentheses) in the indicated clinicophathological categories are shown. *p*-value, Chi-square test *p*-values.

**Additional file 2: Figure S2.** (related to Fig. 3.). The five selected cancer cell lines and their ILEI KD derivatives show similar sensitivity towards the c-MET inhibitors PHA665752 and savolitinib as for crizotinib. **a, b** Viability of NCI-H1993, NCI-H441, MKN45, OE33 and SKBR3 cells towards increasing concentrations of the c-MET inhibitors PHA665752 (a) and savolitinib (b). Fitting curve was normalized to untreated control condition. Dashed line marks IC50.

**Additional file 3: Figure S3**. (related to Fig. 5.). The selective c-MET inhibitor savolitinib reproduces the effects of crizotinib on ILEI secretion, HGF-induced expression of MMPs and E-cadherin expression. **a** Western blot analysis of ILEI secretion and expression, and c-MET activity and expression in the five selected cell lines (parental) and their control (shCont) and ILEI KD (sh261 and sh506) derivatives after savolitinib (1 μM) treatment for 24 h. **b** qPCR analysis of MMP-9 (for NCI-H441, MKN45 and OE33) and MMP-2 (for NCI-H1993 and SKBR3) mRNA expression in control (shCont) and ILEI KD (shILEI) cells after 24 h of HGF treatment (40 ng/ml) in the absence or presence of savolitinib (1 μM). Data are normalized as fold change to untreated control cells. Error bars represent SEM of three independent experiments. Statistical significance was determined by one-way ANOVA. **c** qPCR analysis of E-cadherin mRNA expression (CDH1) in NCI-H1993, NCI-H441, MKN45, OE33 and SKBR3 control (shCont) and ILEI KD (shILEI) cells treated or non-treated with savolitinib (1 μM) for 24 h. Data are normalized as fold change to untreated control cells. Error bars represent SEM of three independent experiments. Statistical significance was determined by one-way ANOVA and marked with asterisks (*p < 0.05; **p < 0.01; ***p < 0.001, p < 0.001; ****p < 0.0001).

**Additional file 4: Figure S4.** (related to Fig. 6.). The effect of ILEI KD and crizotinib treatment on ILEI expression, c-Met expression and phosphorylation and tumor vessel formation. **a, g** Representative images of ILEI IHC on NCI-H441 (a) and NCI-H1993 (g) shCont and shILEI tumor sections of vehicle or Crizo treated mice. Scale bar, 100 μm. **b, h** Representative images of cMET IHC on NCI-H441 (b) and NCI-H1993 (h) shCont and shILEI tumor sections of vehicle or Crizo treated mice. Scale bar, 100 μm. **c, i** Representative images of phospho-cMET IHC on NCI-H441 (c) and NCI-H1993 (i) shCont and shILEI tumor sections of vehicle or Crizo treated mice. Scale bar, 100 μm. **d, j** Quantification of cMET activation in NCI-H441 (d) and NCI-H1993 (j) shCont and shILEI tumors of vehicle or Crizo treated mice determined as percentage of phospho-cMET (pMET) positive tumor cells on IHC stained tissue sections. Note, in NCI-H1993 xenografts crizotinib was withdrawn 11 days before time point of analysis. Error bars represent SEM. Statistical significance was determined by one-way ANOVA. **e, f, k, l** Quantification of tumor vessels in NCI-H441 (e, f) and NCI-H1993 (k, l) shCont and shILEI tumors of vehicle or Crizo treated mice by average vessel density (e, k) and average vessel size (f, l) as determined from CD31 stained tumor tissue sections.

## Data Availability

GISTIC 2.0 copy number calls were extracted from the TCGA datasets BRCA, HNSC, LUAD and LUSC using the download function of cBio database (http://www.cbioportal.org/). Other datasets generated and/or analyzed during the current study are available from the corresponding author on reasonable request.

## Abbreviations

ANOVA: Analysis of variance
CM: Conditioned medium
CN: Copy number
ECL: Enhanced chemiluminescent
DAB: 3,3′-diaminobenzidine
DAPI: 4′,6-diamidino-2-phenylindole
EGFR: Epidermal growth factor receptor
EMT: Epithelial-mesenchymal transition
EMVI: Extramural venous invasion
FCS: Fetal calf serum
FGFR1: Fibroblast growth factor receptor 1
HER: Human epidermal growth factor receptor
HGF: Hepatocyte growth factor
H&E: Hematoxylin and eosin
hnRNP E1: Heterogeneous nuclear ribonucleoprotein E1
ILEI: Interleukin-like EMT inducer
KD: Knock-down
LIFR: Leukemia inhibitory factor receptor
LUAD: Lung adenocarcinoma
LUSC: Lung squamous cell carcinoma
MMP: Matrix metalloproteinase
MTT: 3-(4,5-dimethylthiazol-2-yl)-2,5-diphenyltetrazolium bromide
NSCLC: Non-small-cell lung carcinoma
PBS: Phosphate-buffered saline
SCID: Severe combined immunodeficiency disease
SEM: Standard error of the mean
STAT3: Signal transducer and activator of transcription 3
TGF-β: Transforming growth factor beta
uPAR: Urokinase-type plasminogen activator receptor
VEGF: Vascular endothelial growth factor
VEGFR: VEGF receptor
