# Supplementary figures and images for: The FAM3C locus that encodes interleukin-like EMT inducer (ILEI) is frequently co-amplified in MET-amplified cancers and contributes to invasiveness
Source: J Exp Clin Cancer Res. 2021 Feb 17;40:69. doi: 10.1186/s13046-021-01862-5 (PMC7890988; doi:10.1186/s13046-021-01862-5)

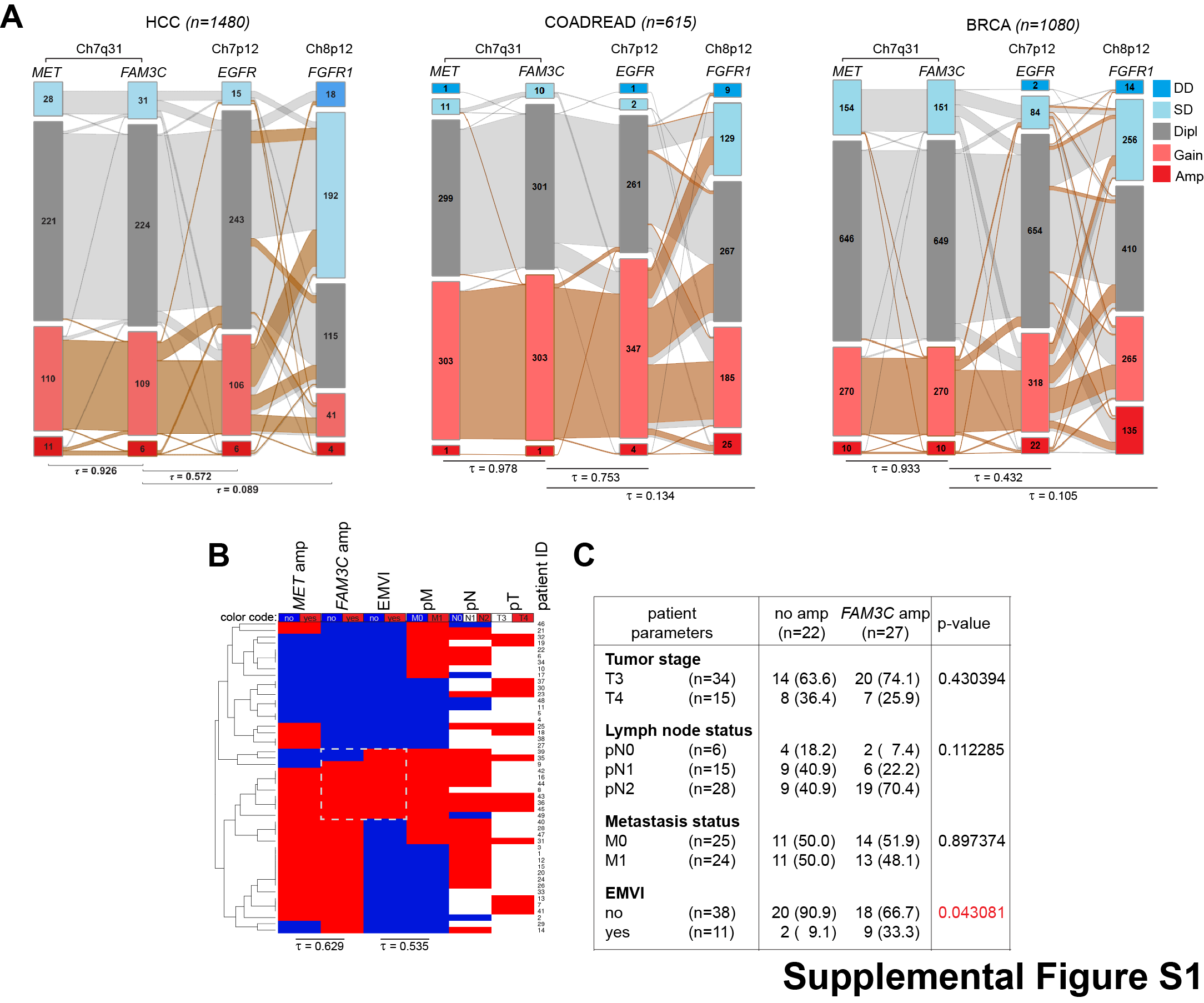

Supplement: Supplementary file 1 — Additional file 1: Figure S1. (related to Fig. 1.). FAM3C and MET gene copy numbers are tightly linked in human hepatocellular, colorectal and breast cancers with FAM3C amplification correlating with venous invasion in colorectal cancer patients. a Summary of MET, FAM3C, EGFR and FGFR1 copy number calls extracted from hepatocellular carcinoma LIHC (left panel), colorectal adenocarcinoma COADREAD (mid panel) and breast carcinoma BRCA (right panel) TCGA datasets. Genomic localization is marked above the genes. GISTIC 2.0 values are shown: − 2, deep deletion (DD); − 1, shallow deletion (SD); 0, diploid (Dipl); 1, gain; 2, amplification (Amp). Numbers within the boxes refer to the number of tumors within a group. Orange connection lines indicate tumor samples with FAM3C gain or amplification. Correlation was calculated using Kendall’s tau-b test. b Heatmap with hierarchical clustering of FAM3C and MET gene amplification determined by Taqman qPCR and of extramural venous invasion (EMVI), tumor stage (pT), lymph node status (pN) and metastasis status (pM) clinicopathological parameters extracted from 49 primary colon carcinoma patients. Calculated copy numbers of 3 or higher were considered as gene amplification. Correlation was calculated using Kendall’s tau-b test. c Correlation of FAM3C gene amplification status with established clinicopathological parameters of the 49 human colorectal cancer patients shown in panel b. The number of patients (n) and their relative frequencies (in %; numbers in parentheses) in the indicated clinicophathological categories are shown. p-value, Chi-square test p-values. [file 13046_2021_1862_MOESM1_ESM.tif]

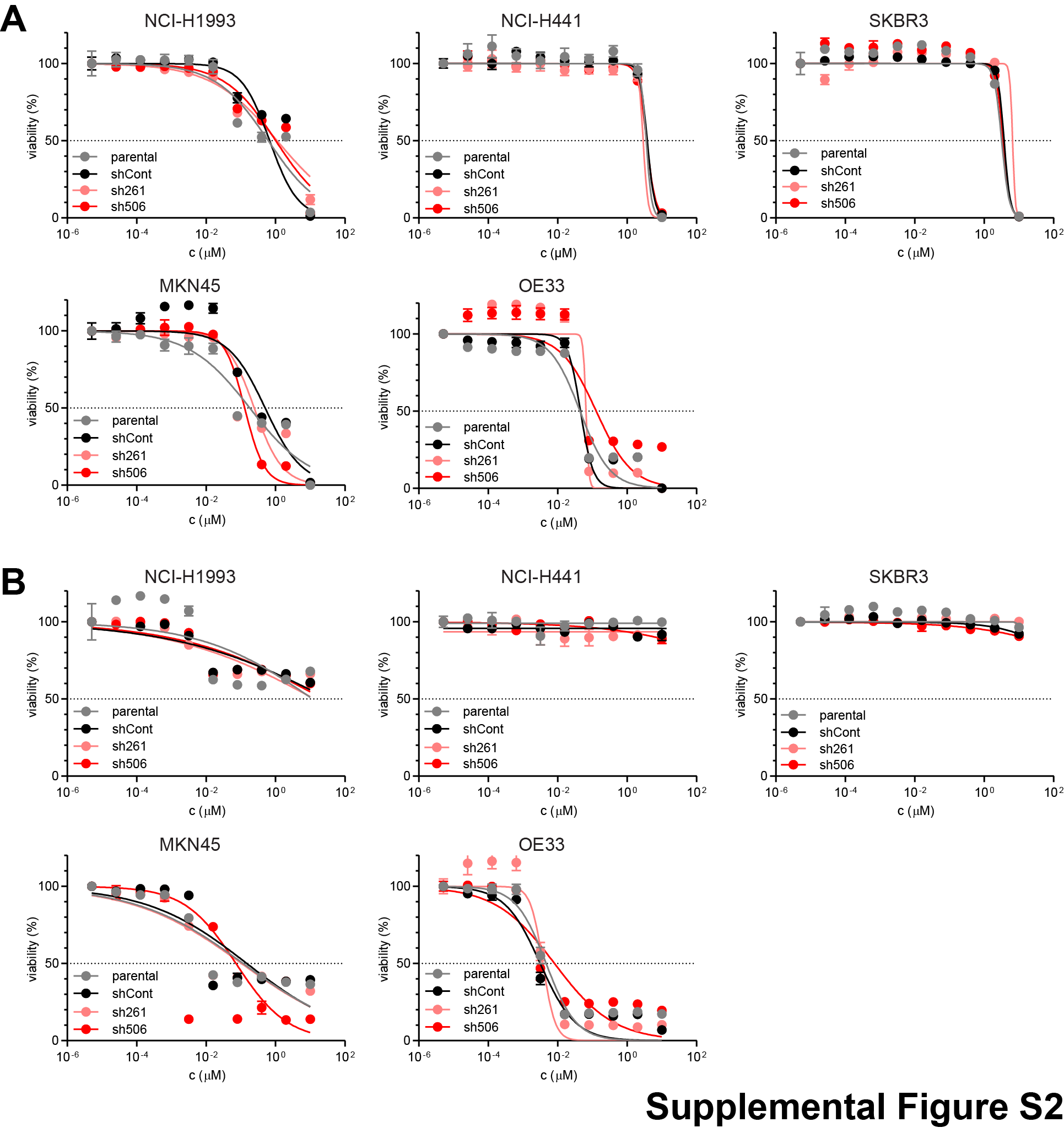

Supplement: Supplementary file 2 — Additional file 2: Figure S2. (related to Fig. 3.). The five selected cancer cell lines and their ILEI KD derivatives show similar sensitivity towards the c-MET inhibitors PHA665752 and savolitinib as for crizotinib. a, b Viability of NCI-H1993, NCI-H441, MKN45, OE33 and SKBR3 cells towards increasing concentrations of the c-MET inhibitors PHA665752 (a) and savolitinib (b). Fitting curve was normalized to untreated control condition. Dashed line marks IC50. [file 13046_2021_1862_MOESM2_ESM.tif]

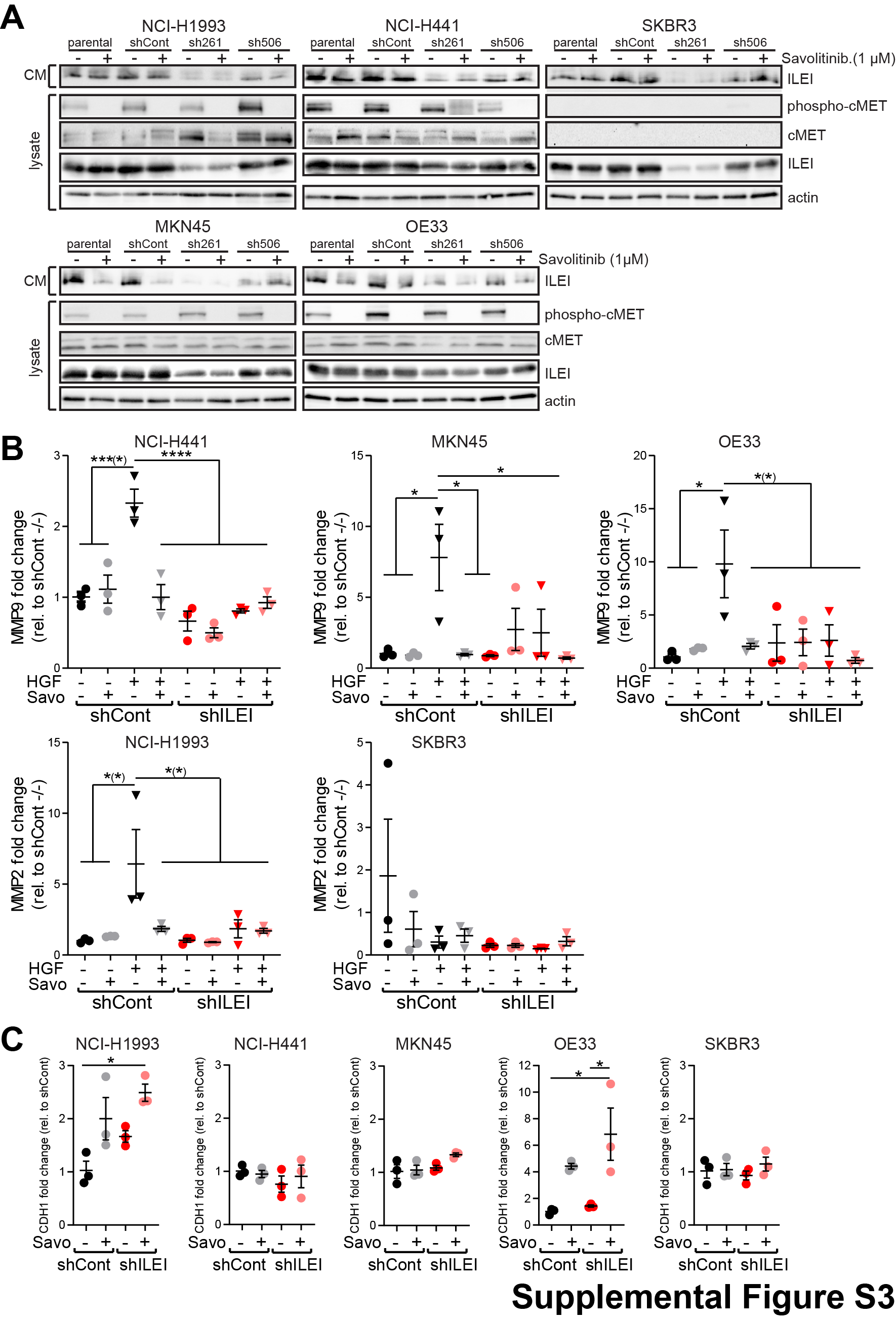

Supplement: Supplementary file 3 — Additional file 3: Figure S3. (related to Fig. 5.). The selective c-MET inhibitor savolitinib reproduces the effects of crizotinib on ILEI secretion, HGF-induced expression of MMPs and E-cadherin expression. a Western blot analysis of ILEI secretion and expression, and c-MET activity and expression in the five selected cell lines (parental) and their control (shCont) and ILEI KD (sh261 and sh506) derivatives after savolitinib (1 μM) treatment for 24 h. b qPCR analysis of MMP-9 (for NCI-H441, MKN45 and OE33) and MMP-2 (for NCI-H1993 and SKBR3) mRNA expression in control (shCont) and ILEI KD (shILEI) cells after 24 h of HGF treatment (40 ng/ml) in the absence or presence of savolitinib (1 μM). Data are normalized as fold change to untreated control cells. Error bars represent SEM of three independent experiments. Statistical significance was determined by one-way ANOVA. c qPCR analysis of E-cadherin mRNA expression (CDH1) in NCI-H1993, NCI-H441, MKN45, OE33 and SKBR3 control (shCont) and ILEI KD (shILEI) cells treated or non-treated with savolitinib (1 μM) for 24 h. Data are normalized as fold change to untreated control cells. Error bars represent SEM of three independent experiments. Statistical significance was determined by one-way ANOVA and marked with asterisks (*p < 0.05; **p < 0.01; ***p < 0.001, p < 0.001; ****p < 0.0001). [file 13046_2021_1862_MOESM3_ESM.tif]

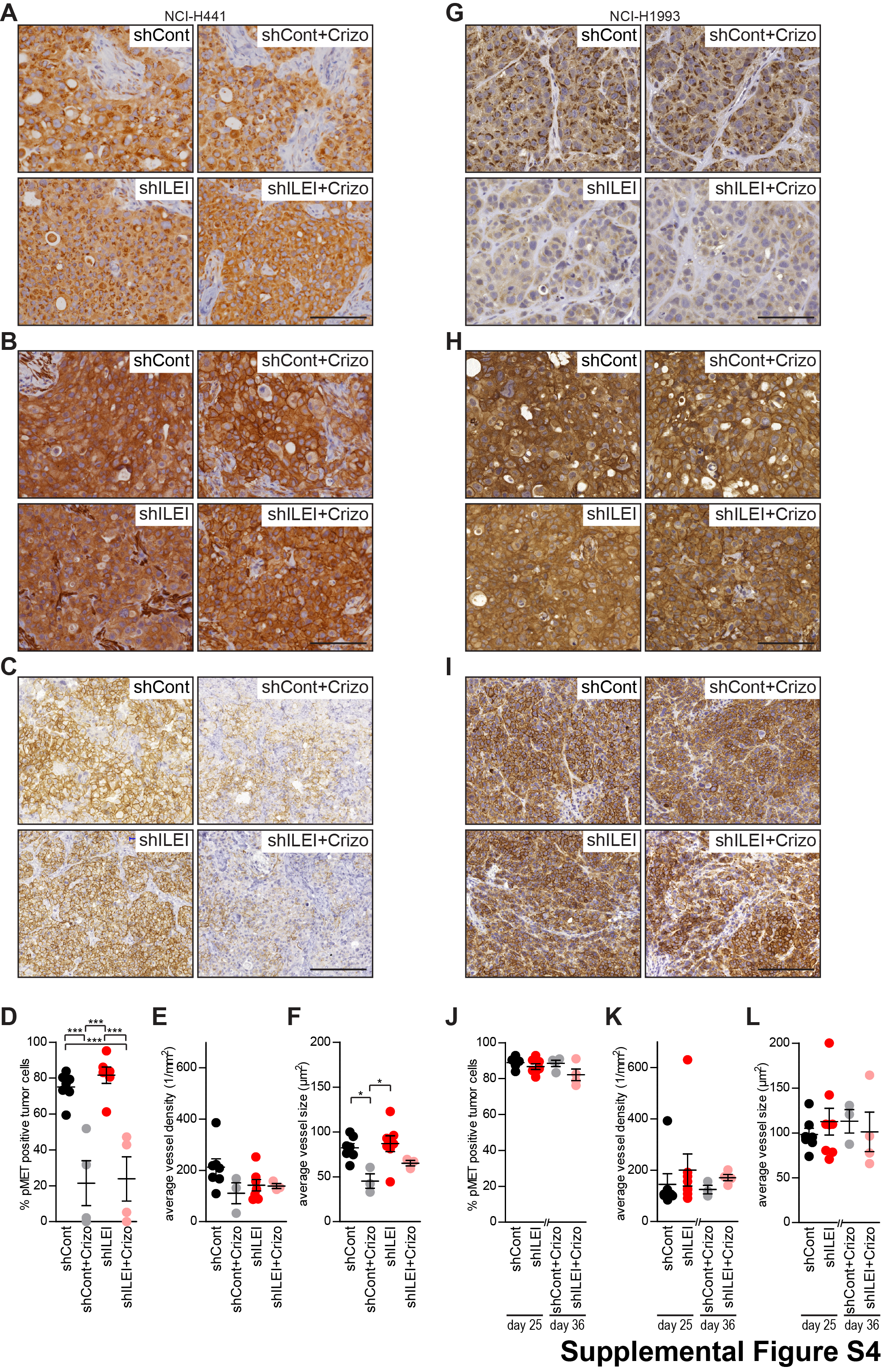

Supplement: Supplementary file 4 — Additional file 4: Figure S4. (related to Fig. 6.). The effect of ILEI KD and crizotinib treatment on ILEI expression, c-Met expression and phosphorylation and tumor vessel formation. a, g Representative images of ILEI IHC on NCI-H441 (a) and NCI-H1993 (g) shCont and shILEI tumor sections of vehicle or Crizo treated mice. Scale bar, 100 μm. b, h Representative images of cMET IHC on NCI-H441 (b) and NCI-H1993 (h) shCont and shILEI tumor sections of vehicle or Crizo treated mice. Scale bar, 100 μm. c, i Representative images of phospho-cMET IHC on NCI-H441 (c) and NCI-H1993 (i) shCont and shILEI tumor sections of vehicle or Crizo treated mice. Scale bar, 100 μm. d, j Quantification of cMET activation in NCI-H441 (d) and NCI-H1993 (j) shCont and shILEI tumors of vehicle or Crizo treated mice determined as percentage of phospho-cMET (pMET) positive tumor cells on IHC stained tissue sections. Note, in NCI-H1993 xenografts crizotinib was withdrawn 11 days before time point of analysis. Error bars represent SEM. Statistical significance was determined by one-way ANOVA. e, f, k, l Quantification of tumor vessels in NCI-H441 (e, f) and NCI-H1993 (k, l) shCont and shILEI tumors of vehicle or Crizo treated mice by average vessel density (e, k) and average vessel size (f, l) as determined from CD31 stained tumor tissue sections. [file 13046_2021_1862_MOESM4_ESM.tif]
